# A Possible Recently Identified Evolutionary Strategy Using Membrane-Bound Vesicle Transfer of Genetic Material to Induce Bacterial Resistance, Virulence and Pathogenicity in *Klebsiella oxytoca*

**DOI:** 10.3390/ijms27020988

**Published:** 2026-01-19

**Authors:** Yahaira de Jesús Tamayo-Ordóñez, Ninfa María Rosas-García, Juan Manuel Bello-López, María Concepción Tamayo-Ordóñez, Francisco Alberto Tamayo-Ordóñez, Claudia Camelia Calzada-Mendoza, Benjamín Abraham Ayil-Gutiérrez

**Affiliations:** 1 Laboratorio de Biotecnología Ambiental, Centro de Biotecnología Genómica, Instituto Politécnico Nacional, Reynosa 88710, Tamaulipas, Mexico; nrosas@ipn.mx; 2 División de Investigación, Hospital Juárez de México, Mexico City 07760, Mexico; juanmanuelbello81@hotmail.com; 3 Laboratorio de Ingeniería Genética, Departamento de Biotecnología, Facultad de Ciencias Químicas, Universidad Autónoma de Coahuila, Saltillo 25280, Coahuila, Mexico; mtamayo@uadec.edu.mx; 4 Facultad de Química, Universidad Autónoma del Carmen, Calle 56 N. 4, Av. Concordia Col. Benito Juárez, Carmen City 24180, Campeche, Mexico; ftamayo@pampano.unacar.mx; 5 Sección de Estudios de Posgrado e Investigación, Escuela Superior de Medicina, Instituto Politécnico Nacional, Mexico City 11340, Mexico; cccalzadam@yahoo.com.mx; 6 SECIHTI-Centro de Biotecnología Genómica, Instituto Politécnico Nacional, Biotecnología Vegetal, Reynosa 88710, Tamaulipas, Mexico

**Keywords:** *Klebsiella oxytoca*, outer membrane vesicles, comparative genomics, pangenome analysis, mobile genetic elements, antimicrobial resistance, genome based inference

## Abstract

*Klebsiella oxytoca* has emerged as an important opportunistic pathogen in nosocomial infections, particularly during the COVID-19 pandemic, due to its capacity to acquire and disseminate resistance and virulence genes through horizontal gene transfer (HGT). This study presents a genome-based comparative analysis of *K. oxytoca* within the genus *Klebsiella*, aimed at exploring the evolutionary plausibility of outer membrane vesicle (OMV) associated processes in bacterial adaptation. Using publicly available reference genomes, we analyzed pangenome structure, phylogenetic relationships, and the distribution of mobile genetic elements, resistance determinants, virulence factors, and genes related to OMV biogenesis. Our results reveal a conserved set of envelope associated and stress responsive genes involved in vesiculogenic pathways, together with an extensive mobilome and resistome characteristic of the genus. Although these genomic features are consistent with conditions that may favor OMV production, they do not constitute direct evidence of functional OMV mediated horizontal gene transfer. Instead, our findings support a hypothesis generating evolutionary framework in which OMVs may act as a complementary mechanism to established gene transfer routes, including conjugation, integrative mobile elements, and bacteriophages. Overall, this study provides a genomic framework for future experimental and metagenomic investigations into the role of OMV-associated processes in antimicrobial resistance dissemination and should be interpreted as a recently identified evolutionary strategy inferred from genomic data, rather than a novel or experimentally validated mechanism.

## 1. Introduction

*Klebsiella oxytoca* is an opportunistic Gram-negative bacterium of the Enterobacterales group, frequently associated with nosocomial infections such as pneumonia, urinary tract infections, bacteremia, and sepsis [1]. Historically, it has been less studied than *Klebsiella pneumoniae*, but its clinical importance has grown in recent years due to the emergence of multi-resistant strains, producers of extended-spectrum β-lactamase (ESBL) and carbapenemase [2]. This situation worsened during the COVID-19 pandemic, in which prolonged use of antibiotics, mechanical ventilation, and prolonged hospital stays favored the emergence of severe bacterial coinfections, many of them caused by *Klebsiella* spp. [3]. In the case of *K*. *oxytoca*, outbreaks have been documented in intensive care units, associated with high morbidity and mortality in immunocompromised patients [4].

In Mexico, factors such as hospital overcrowding, lack of strict hygiene protocols, and indiscriminate use of antibiotics have facilitated the selection and spread of resistant strains [4]. The origin of *K. oxytoca* outbreaks is often related to lapses in the sterilization of medical equipment, cross-contamination in patient management, and failures in infection control measures. In several hospital units in the country, outbreaks of *K. oxytoca* infections in neonates were detected, associated with contamination of intravenous solutions and catheters [5]. These events underscore the need to strengthen epidemiological surveillance, implement strict hygiene programs, and optimize the use of antibiotics in the hospital environment.

Currently, *K. oxytoca* remains a persistent threat in clinical settings, not only due to its multidrug resistance, but also due to its ability to acquire and transfer resistance and virulence genes through horizontal transfer mechanisms. Among these mechanisms, the role of outer membrane vesicles (OMVs) has begun to be recognized as an emerging and relevant pathway for the dissemination of mobile genetic elements (MGEs), such as plasmids, integrons, transposons, and insertion sequences [6]. In Gram-negative bacteria, outer membrane vesicles (OMVs) play a fundamental role in diverse biological processes. These structures are involved in virulence, horizontal gene transfer (HGT), export of cellular metabolites, phage infection, intercellular communication, and modulation of the host immune response [7].

Outer membrane vesicles (OMVs)—a major class of bacterial extracellular vesicles (EVs) produced by Gram-negative bacteria—are among the most intensively studied structures due to their broad biological relevance and potential biomedical applications. In Gram-negative bacteria, EVs have been investigated for their involvement in intercellular communication, modulation of host immune responses, transport of bioactive compounds, vaccine development, and their proposed contribution to antimicrobial resistance and genetic exchange [6,8,9]. Two main mechanisms underlying EV formation in Gram-negative bacteria have been described: (i) active vesicle release from viable cells, resulting in the production of classical OMVs derived from the outer membrane; and (ii) vesicle formation associated with cell envelope remodeling or lytic processes, giving rise to less frequent vesicle subpopulations such as outer–inner membrane vesicles (OIMVs) or burst membrane vesicles (BMVs) [6].

OMVs are lipid structures shed from the outer membrane of Gram-negative bacteria [6]. These vesicles transport fragments of the periplasm and cell wall, as well as various components of the outer membrane itself, including lipids, proteins, and, in some cases, nucleic acids. In certain bacteria, an increased capacity to produce OMVs has been shown to be a generalized response to cellular stress, induced by the accumulation of toxic metabolites or misfolded proteins. This local accumulation generates an invagination of the outer membrane, resulting in the extracellular release of OMVs containing these potentially harmful byproducts [7,8].

There is a subpopulation of bacterial extracellular vesicles (EVs) known as outer–inner membrane vesicles (OIMVs). These structures are characterized by the presence of two lipid bilayers, corresponding to the inner and outer bacterial membranes. In contrast to classical OMVs, OIMVs can encapsulate not only periplasmic and outer membrane components but also molecules originating from the inner membrane and cytoplasmic compartment, including ATP, nucleic acids, and other cytoplasmic macromolecules. Experimental studies have demonstrated that several Gram-negative bacterial species are capable of producing OIMVs and that this vesicle subpopulation may contain DNA and RNA cargo [7]. The biological relevance of OMVs produced by pathogenic bacteria is primarily associated with their capacity to transport a wide range of molecular cargos, including virulence-associated proteins, adhesins, toxins, and immunomodulatory factors. Representative examples include OmpA-family proteins (e.g., OprF) in *Pseudomonas aeruginosa* and the vacuolating cytotoxin VacA and the HopA porin in *Helicobacter pylori* [10]. Host–pathogen interactions have been shown to influence OMV release and cargo composition. For instance, *H. pylori* OMVs have been reported to contain adhesins such as sialic acid–binding adhesin (SabA) and blood group antigen–binding adhesin (BabA), together with additional virulence factors that may contribute to host cell interaction and immune modulation [10,11]. The uptake of OMVs by host cells occurs through multiple endocytic pathways, including clathrin-mediated, caveolin-dependent, and lipid-raft-associated mechanisms. These processes are influenced by physicochemical properties of the vesicles, such as lipopolysaccharide (LPS) structure and membrane composition, rather than by active targeting mechanisms [12].

Bacterial resistance to antibiotics represents a major public health challenge, further underscored by estimates indicating that more than 70% of pathogens responsible for nosocomial infections exhibit resistance to at least one commonly used antimicrobial agent. In addition, antimicrobial resistance is projected to cause cumulative losses of up to USD 100 trillion in the global gross domestic product (GDP) by 2050, positioning it among the most significant global economic threats [6,13]. Within this context, bacterial extracellular vesicles (EVs)—including outer membrane vesicles (OMVs) and burst membrane vesicles (BMVs)—have been implicated in antibiotic-resistance-associated phenomena in selected experimental systems. Reported mechanisms include: (a) vesicle-mediated sequestration of antimicrobial compounds acting as decoys; (b) association of resistance determinants within vesicular cargo; (c) contribution to antibiotic extrusion through vesicle-associated efflux components; and (d) extracellular inactivation of antibiotics via vesicle-associated degradative enzymes [14,15,16]. Importantly, these mechanisms have been demonstrated primarily in specific model organisms and experimental settings and should not be generalized across bacterial species without direct functional validation.

In this framework, genomic analyses of *K. oxytoca* provide an opportunity to examine the evolutionary plausibility of OMV-associated processes within a broader context of bacterial adaptation. This study integrates four complementary in silico approaches: (1) genome-wide identification of genes associated with OMV biogenesis and envelope maintenance; (2) pangenome reconstruction of the *Klebsiella* genus; (3) comparative and phylogenetic analyses of proteins previously implicated in vesicle-associated processes; and (4) comprehensive characterization of the mobilome, resistome, and virulome. Notably, this work is based exclusively on genomic inference and does not include experimental isolation of vesicles, validation of nucleic acid cargo, or vesicle uptake assays. The evolutionary strategy discussed here is not proposed as a novel phenomenon, but rather as a recently identified and computationally inferred strategy that may have operated in bacterial populations over extended evolutionary timescales.

Accordingly, the OMV-related model proposed here should be regarded as a hypothesis-generating evolutionary framework, rather than as direct evidence of functional OMV-mediated horizontal gene transfer. By explicitly contrasting genomic features potentially associated with OMVs with well-established HGT mechanisms and by clearly acknowledging the methodological limitations of an in silico approach, this study provides a rigorous genomic baseline for future experimental, functional, and metagenomic investigations aimed at clarifying the role of bacterial extracellular vesicles in genome evolution and antimicrobial resistance.

## 2. Results

### 2.1. Klebsiella Pangenome and Its Potential for Gene Mobilization via OMVs

**Figure 1:**
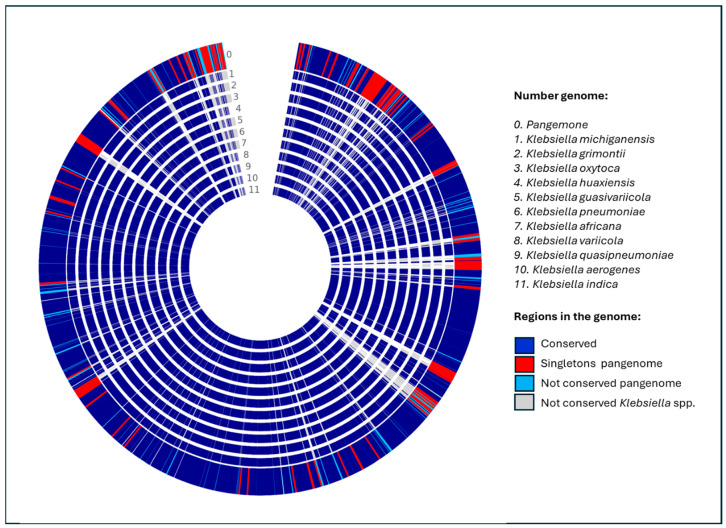
Pangenome representation based on 11 reference genomes of selected *Klebsiella* species. The figure illustrates the observed distribution of gene clusters across the analyzed dataset. Regions conserved among all included genomes represent the core gene set and are shown in dark blue. Genes unique to individual genomes are indicated in red. Gene clusters present in multiple but not all genomes are shown in light blue, whereas absent regions are depicted in gray. This representation is descriptive and comparative in nature and does not aim to infer genus-wide pangenome dynamics beyond the analyzed reference genomes. The analyzed genomes are labeled as follows: 0, total gene cluster set; 1, *K. michiganensis*; 2, *K. grimontii*; 3, *K. oxytoca*; 4, *K. huaxiensis*; 5, *K. guasivariicola*; 6, *K. pneumoniae*; 7, *K. africana*; 8, *K. variicola;* 9, *K. quasipneumoniae*; 10, *K. aerogenes*; 11, *K. indica*.

The pangenome of 11 representative species of the genus *Klebsiella* was constructed by comparative analysis of whole genomes (Figure 1 and Table 1). The resulting pangenome comprised a total of 65,187 coding genes, of which 60,277 genes clustered into homologous families that make up the core gene set, i.e., genes conserved in all analyzed species. This core gene set represents approximately 92% of the total, indicating a high evolutionary conservation associated with essential functions for cell survival and basic physiological maintenance [17,18]. The high proportion of the core genome (~92%) observed in this analysis should be interpreted with caution. Given the limited number of representative genomes and the inclusion of mostly single genomes per species, this value is likely inflated and does not reflect the true core genome size at the genus level. In contrast, 4910 unique genes belonging to singleton families, specific to each species, were identified. These genes could be related to particular adaptations, including differences in virulence, antibiotic resistance, and other relevant phenotypic characteristics [19,20,21]. The present dataset does not capture intraspecies genomic diversity, particularly for clinically relevant species such as *K. oxytoca* and *K. pneumoniae*. As a result, strain-specific variation, accessory gene dynamics, and lineage-dependent adaptations are not represented in this pangenome reconstruction [22].

Analysis of homologous families among the 11 species (Table 2) revealed high genomic conservation within certain clades, particularly between *K. pneumoniae*, *K. quasipneumoniae*, and *K. variicola* (e.g., G8–G9: 4090 families; G8–G11: 4184 families), suggesting either a close phylogenetic relationship or a recent history of gene exchange. In contrast, more divergent species such as *K. indica* (G5) displayed fewer shared homologous families (G5–G4: 3457; G5–G9: 3208), indicating either a lower conservation of the gene repertoire or limited involvement in horizontal transfer events. These differences can be explained, in part, by the action of mobile genetic elements (MGEs), such as plasmids, transposons, integrons and bacteriophages, which mediate the acquisition and spread of genes associated with virulence, antimicrobial resistance and specialized metabolism [23,24]. Furthermore, the high genomic similarity observed between species such as *K. michiganensis* (G6), *K. oxytoca* (G7) and *K. grimontii* (G3), which share more than 4400 gene families in some pairs, suggests a high gene flow, possibly facilitated by cohabitation in similar niches or hospitable environments, where MGEs act as vectors of genetic adaptation [25].

In this context, OMVs emerge as potential vehicles for the transfer of genetic material between bacteria, including genes associated with resistance and virulence [26]. Recent studies have shown that OMVs can transport DNA, RNA and functional proteins, thus contributing to the plasticity of the bacterial genome and the evolution of the pangenome [27,28]. The combination of an open pangenome and a high activity of EGMs suggests that *Klebsiella* spp. possess a remarkable evolutionary potential, with significant clinical implications in the emergence of multidrug-resistant and hypervirulent clones. Gene content analysis revealed that *K. oxytoca* shares a large number of genes related to antimicrobial resistance (AMR) and OMV production. Key genes detected include blaCTX-M-15, blaSHV-11, oqxA, oqxB, fosA, marA, acrB, and tolC, which belong to families such as class A β-lactamases, RND-type efflux pumps, and fosfomycin resistance proteins [29,30]. These genes confer resistance to several classes of antibiotics, including β-lactams, fluoroquinolones, macrolides, and fosfomycin, through mechanisms such as enzymatic hydrolysis, active efflux, and antimicrobial target modification [14,31]. It is important to note that these observations are based exclusively on genomic associations. The presence of OMV-related genes and mobile genetic elements does not constitute direct evidence of functional OMV-mediated gene transfer but rather supports an inference-based framework consistent with evolutionary plausibility.

The detection of the potential for mobilization of resistance genes by OMVs in *K. oxytoca* is relevant. Several studies have shown that OMVs can act as efficient vehicles for horizontal transfer, encapsulating and protecting genetic material, including resistance and virulence genes [32,33]. In this context, the genes identified in *K. oxytoca* could be disseminated not only by classical mechanisms such as plasmids and integrons but also through OMVs, which represents an emerging route of gene mobilization with clinical implications. The high genomic similarity of *K. oxytoca* with other species of the genus, combined with its mobile genetic load and its participation in horizontal transfer mechanisms, reinforce its role as a reservoir and disseminator of resistance and virulence genes, especially in hospital settings [34,35]. The genomic co-occurrence of resistance genes, mobile genetic elements, and OMV-associated loci suggests conditions that may be compatible with vesicle-associated mobilization. However, these data do not demonstrate active gene transfer mediated by OMVs and should be interpreted as correlational rather than mechanistic.

### 2.2. Genes Involved in the Biogenesis and Release of Outer Membrane Vesicles in K. oxytoca

Genomic analysis of *K. oxytoca* identified a set of 32 genes commonly associated in the literature with OMV biogenesis, envelope maintenance, and stress responses in Gram-negative bacteria. It is important to emphasize that these genes are broadly conserved and primarily fulfill essential cellular functions; therefore, their presence should not be interpreted as direct evidence of enhanced vesiculation or OMV-mediated HGT. In this study, these genes were grouped into five functional categories based on annotated roles: (1) outer membrane stability and protein assembly; (2) periplasmic quality control and stress response; (3) lipopolysaccharide (LPS) biosynthesis and remodeling; (4) peptidoglycan remodeling and cell envelope dynamics; and (5) lipid metabolism and oxidative stress adaptation (Table 3 and Table S1). Together, these categories encompass structural, metabolic, and regulatory functions that contribute to envelope integrity and physiological adaptation.

Genes such as ompA, lpp, pal, tolA, tolB, tolR, bamA, and bamB were identified as components of conserved envelope-associated systems, including the Tol–Pal complex and the β-barrel assembly machinery (BAM). These systems are essential for anchoring the outer membrane to the peptidoglycan layer and for the proper insertion and assembly of outer membrane proteins. Although disruption of some of these genes—particularly ompA—has been associated in other Gram-negative bacteria with increased vesiculation due to envelope destabilization, such observations derive from experimental studies in diverse model organisms and do not imply a similar phenotype in *K. oxytoca* under the conditions analyzed here [35,36]. Additional genes, including degP, surA, nlpI, pmrA, pmrB, phoP, and phoQ, were identified and are known to participate in periplasmic protein quality control, envelope homeostasis, and stress-responsive regulatory pathways. These systems are involved in sensing and responding to oxidative, osmotic, and antimicrobial stress, processes that have been correlated with changes in vesiculation dynamics in Gram-negative bacteria. However, their presence in the *K. oxytoca* genome reflects conserved stress-adaptation mechanisms rather than functional evidence of OMV-mediated genetic transfer (Table 3) [37,38].

**Figure 2:**
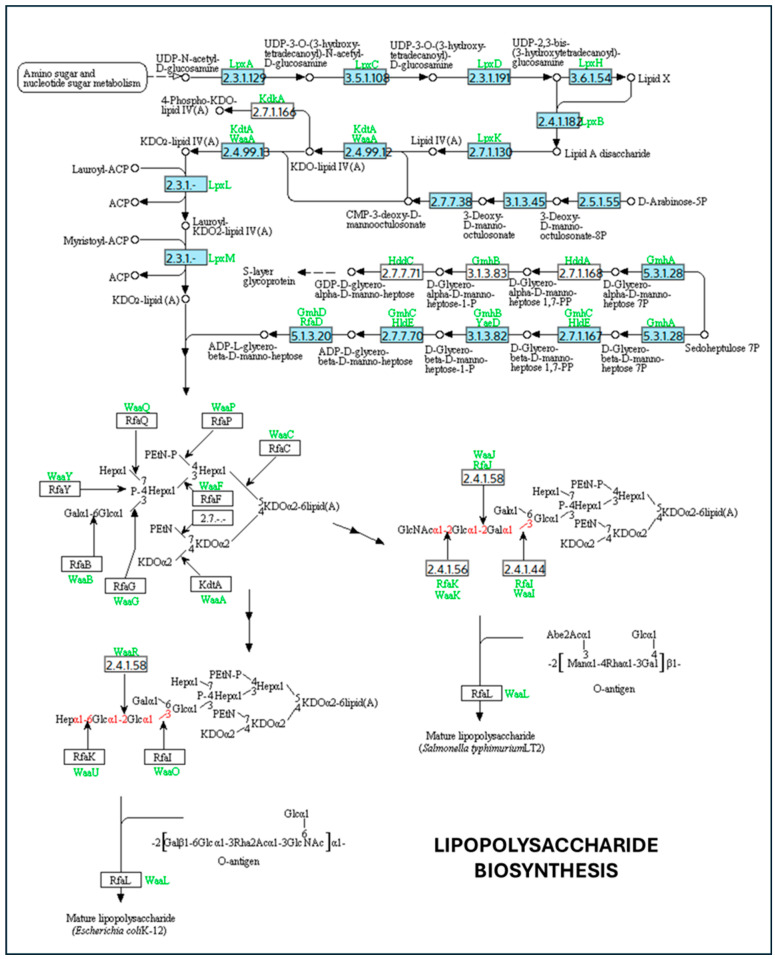
Lipopolysaccharide (LPS) biosynthesis pathway reconstructed from the *Klebsiella oxytoca* genome. This figure illustrates the reconstructed LPS biosynthesis pathway based on genome annotation of *K. oxytoca*. The pathway includes the main enzymatic steps involved in the synthesis of lipid A, the core oligosaccharide, and the O-antigen. Gene names are shown in green, while enzyme commission (EC) numbers are indicated in blue boxes; these blue boxes indicate genes identified in the *K. oxytoca* genome, confirming the presence of the corresponding enzymatic functions within the pathway. Enzymes or reactions highlighted with red boxes denote key steps of particular relevance identified in this study. Metabolites and intermediates are shown in black. The diagram is intended to provide a visual overview of pathway completeness and organization rather than to depict novel biochemical reactions. LPS biosynthesis is a central process for outer membrane integrity in Gram-negative bacteria and provides the structural basis for membrane remodeling, host–pathogen interactions and OMVs biogenesis. This pathway map therefore supports the functional interpretation of genomic data discussed in this study.

**Figure 3:**
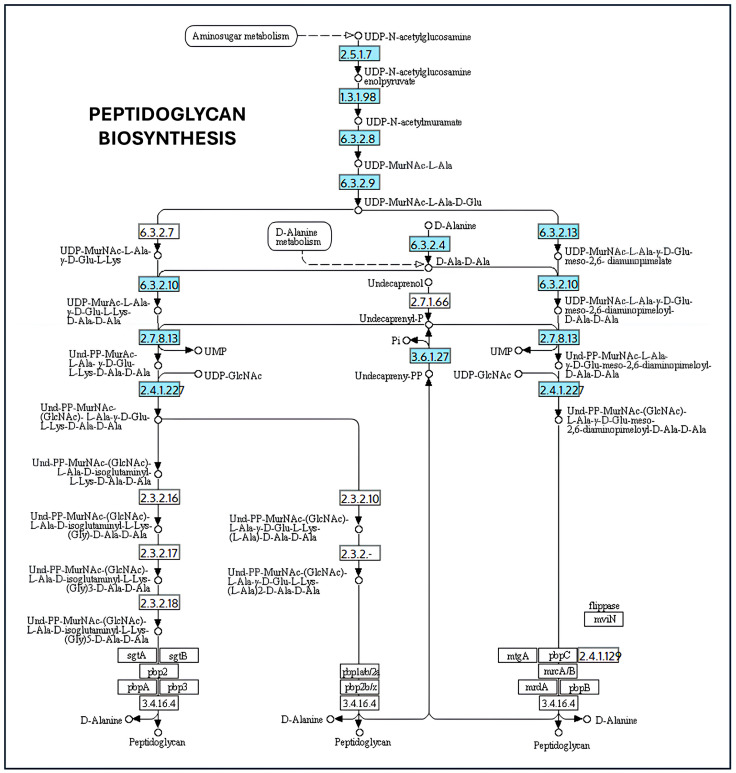
Peptidoglycan biosynthesis pathway reconstructed from the *Klebsiella oxytoca* genome. This figure depicts the reconstructed peptidoglycan biosynthesis pathway based on genome annotation of *K. oxytoca*. The diagram includes the main enzymatic steps involved in the synthesis of peptidoglycan precursors, their coupling to lipid carriers, translocation across the cytoplasmic membrane, and final polymerization and cross-linking of the peptidoglycan network. Blue boxes indicate genes identified in the *K. oxytoca* genome, confirming the presence of the corresponding enzymatic functions within the pathway. The figure is intended to provide a schematic overview of the pathway organization and completeness rather than to represent novel biochemical pathways. Peptidoglycan biosynthesis and remodeling are essential for cell wall integrity in Gram-negative bacteria and play a central role in envelope dynamics, including processes associated with membrane stress adaptation and OMVs biogenesis. This pathway map therefore supports the functional interpretation of the genomic data discussed in this study.

**Figure 4:**
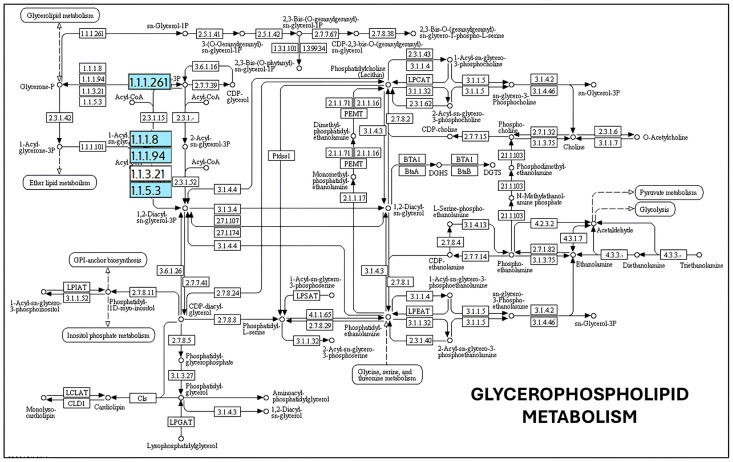
Glycerophospholipid metabolism pathway reconstructed from the *Klebsiella oxytoca* genome. This figure illustrates the reconstructed glycerophospholipid metabolism pathway based on genome annotation of *K. oxytoca*. The pathway encompasses the major enzymatic reactions involved in the synthesis and modification of glycerophospholipids, including the acylation of glycerol-3-phosphate and the formation of key membrane phospholipids such as phosphatidylethanolamine, phosphatidylcholine, and phosphatidylserine. Blue boxes indicate genes identified in the *K. oxytoca* genome, confirming the presence of the corresponding enzymatic activities within this metabolic network. The figure is intended to provide a schematic overview of pathway organization and metabolic potential rather than to depict novel biochemical pathways. Glycerophospholipids constitute fundamental structural components of bacterial membranes and are central to membrane remodeling processes. Their metabolism is therefore relevant to envelope dynamics and may contribute to the biogenesis, composition, and physicochemical properties of OMVs. This pathway map supports the functional interpretation of genomic data discussed in this study.

Genes involved in lipid A and LPS core biosynthesis, including lpxA, lpxB, lpxC, lpxD, lpxH, lpxK, lpxL, lpxM, kdtA (waaA), and gmhA–D, were also detected (Figure 2). LPS is a major structural component of the outer membrane, and alterations in its composition have been reported to influence membrane curvature and vesicle formation in experimental systems. In the present analysis, the identification of these genes indicates the presence of canonical LPS biosynthetic pathways, without implying altered vesiculation phenotypes [39]. Similarly, genes associated with peptidoglycan biosynthesis and remodeling (murA–F, mraY, murG, and uppP) were identified (Figure 3). Peptidoglycan integrity is critical for maintaining cell envelope stability, and perturbations in this pathway have been linked to vesicle release in other bacterial models. In this context, the presence of these genes in *K. oxytoca* is consistent with conserved cell wall biosynthesis rather than with an inferred increase in OMV production [40]. Finally, genes involved in phospholipid metabolism and oxidative stress adaptation, such as gpsA, glpD, plsB, plsC, and selD, were detected (Figure 4). These pathways contribute to membrane lipid homeostasis and redox balance and have been associated with stress responses that may influence vesiculation under specific conditions [41]. Nonetheless, their identification here reflects metabolic and physiological versatility rather than direct evidence of stress-induced OMV overproduction (Table S1). In addition, analysis of the selenocompound metabolic pathway revealed the presence of a single gene encoding selenophosphate synthase (selD; EC 2.7.9.3), while the remaining genes required for complete selenocompound metabolism were absent (Figure S1). This enzyme catalyzes the formation of selenophosphate, an essential precursor for selenoprotein biosynthesis. The partial representation of this pathway suggests a limited capacity for full selenocompound metabolism in *K. oxytoca*, consistent with a restricted but potentially functional selenium utilization system.

### 2.3. Comparative Evolutionary Patterns of Proteins Involved in Outer Membrane Vesicle Mediated Gene Transfer in Klebsiella oxytoca

**Figure 5:**
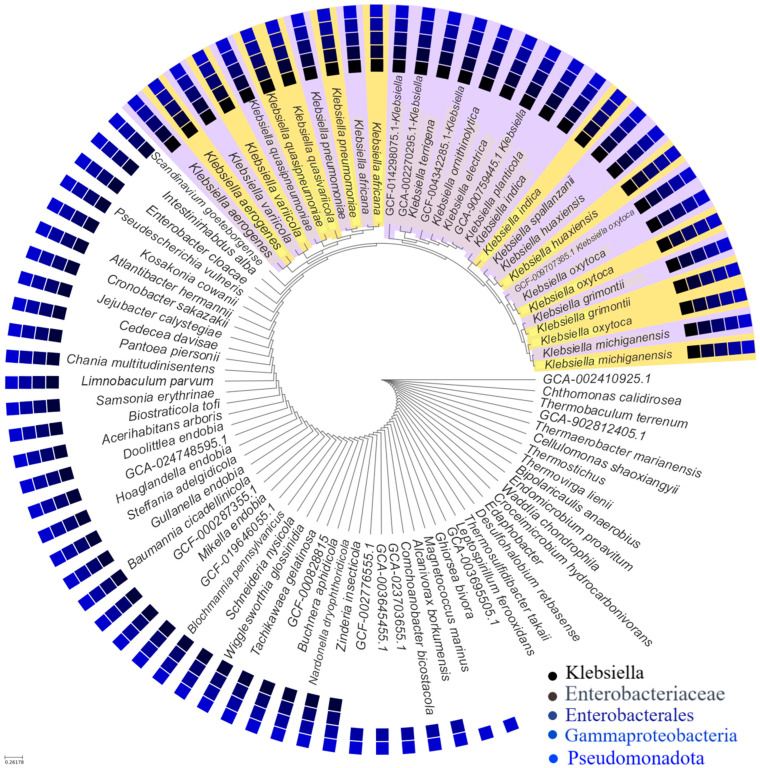
Phylogenomic placement of *Klebsiella* spp. within Enterobacteriaceae and related taxa based on core genome analysis. This figure shows a phylogenetic reconstruction based on the concatenation of core genome genes identified through comparative genomic analysis. The tree was inferred using the maximum likelihood approach implemented in FastTree version 2.1.11 with branch support estimated using the Shimodaira–Hasegawa–like (SH-like) local support values. The phylogeny is intended to provide a genomic context for taxonomic placement and evolutionary relatedness, rather than to propose novel phylogenetic relationships. Genomes analyzed in this study are highlighted in yellow, while closely related lineages within the genus *Klebsiella* are indicated in purple. Representative genomes from multiple taxonomic levels were included to illustrate hierarchical relationships, encompassing members of the phylum Pseudomonadota (Proteobacteria), the class Gammaproteobacteria, the order Enterobacterales, the family Enterobacteriaceae, and species of the genus *Klebsiella*. This phylogenomic framework supports species-level classification and comparative analyses discussed in this study.

A phylogenetic analysis was performed using proteins encoded by genes related to the formation of OMVs, structures involved in HGT processes. To this end, complete genomes of various bacterial species belonging to the phylum Pseudomonadota were selected, with emphasis on the class Gammaproteobacteria and the order Enterobacterales, including pathogenic species of clinical, agricultural, and veterinary importance. The results showed a clearly defined monophyletic clade for species of the genus *Klebsiella*, surrounded by phylogenetically close lineages of the same order, such as *Escherichia*, *Shigella*, *Salmonella* and *Enterobacter* (Figure 5). This hierarchical organization suggests a strong evolutionary conservation of genes involved in OMV biogenesis within *Klebsiella* spp., indicating a selective pressure to maintain these functions associated with survival, virulence, and adaptability in clinical environments [32,42].

To gain a deeper understanding of the functional phylogeny, the TolA, TolB, TolQ, TolR, and Pal genes, components of the Tol-Pal system involved in cell envelope integrity and OMV formation, were individually analyzed. A total of 178 bacterial accessions belonging to 17 genera (*Staphylococcus*, *Mycobacterium*, *Enterococcus*, *Pseudomonas*, *Proteus*, *Yersinia*, *Dickeya*, *Rahnella*, *Pantoea*, *Erwinia*, *Shigella*, *Klebsiella*, *Huaxibacter*, *Enterobacter*, *Lelliottia*, *Salmonella* and *Escherichia*), all known for their pathogenic capacity in humans, plants, or animals, were included. Within the *Klebsiella* clade, *K. oxytoca* clustered closely with other clinically relevant species such as *K. pneumoniae*, *K. quasipneumoniae* and *K. variicola*, sharing high similarity in Tol and Pal protein sequences. Individual dendrograms of TolA and TolB genes (Figures S2 and S3) indicated that *Klebsiella* accessions (*K. variicola*, *K. pneumoniae*, *K. michiganensis*, *K. oxytoca*, K. sp. 141130, *K. aerogenes*, *K. quasipneumoniae*, K. sp. 20 CIP Kleb, *K. michiganensis*, K. sp. ME-303, K. sp. XI-16S-NF2 and *K. pneumoniae* subsp. pneumoniae) share amino acid characteristics closer to *Escherichia* (*E. marmotae* and *E. coli*) and Shigella (*S. flexneri*, *S. boydii* and *S. sonnei*), Enterobacter (*E. mori* and *E. hormaechei*) and *Salmonella enterica* accessions. The clustering of Tol Q and Tol R genes showed greater divergence (Figures S4 and S5); however, it was confirmed that the *Klebsiella* sp. accessions are closer to *Escherichia* sp. *Salmonella* sp. and *Enterobacter* sp.

**Figure 6:**
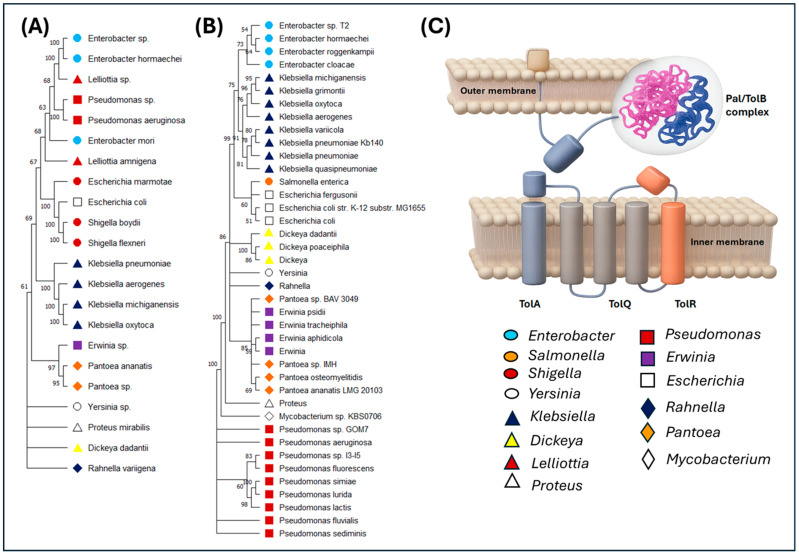
Evolutionary relationships of conserved envelope-associated proteins commonly linked to outer membrane vesicle (OMV) biogenesis in Gram-negative bacteria. (**A**) Phylogenetic trees based on amino acid sequences of the Tol cluster (TolA–TolB–TolQ–TolR). Phylogeny was reconstructed using the Maximum Parsimony method, and clade support was evaluated with 1000 bootstrap replicates. Amino acid sequences were aligned using Alignment Explorer/CLUSTALW, and phylogenetic analyses were performed with MEGA version 11. The analyzed taxa include representatives of *Enterobacter*, *Escherichia*, *Pseudomonas*, *Yersinia*, *Proteus*, *Dickeya*, *Rahnella*, *Erwinia*, *Pantoea*, *Shigella*, *Klebsiella*, and *Lelliottia* spp. *Enterobacter mori* (TolR-UWX94544.1–TolQ-PJD12935.1–TolA-WP_431140005.1–TolB-QWC69181.1), *Enterobacter* sp. (TolR-PVU45235.1–TolQ-ESS60291.1–TolA-WP_431140005.1–TolB-PVU55173.1), *Pseudomonas* sp. (TolR-WAJ36269.1–TolQ-WKE32817.1–TolA-TJY49786.1–TolB-AVZ36123.1), *Escherichia coli* (TolR-PVU59112.1–TolQ-PSY15829.1–TolA-WP_430506710.1–TolB-TKZ09758.1), *Pseudomonas aeruginosa* (TolR-AAC44659.1–TolQ-XCM85803.1–TolA-TJY49786.1–TolB-WAC79344.1), *Yersinia* sp. (TolR-CNF50977.1–TolQ-AJJ25005.1–TolA-WP_427239597.1–TolB-PNM17920.1), *Proteus mirabilis* (TolR-KGA90008.1–TolQ-AWF40160.1–TolA-WP_430266024.1–TolB-BDR97198.1), *Dickeya dadantii* (TolR-UAY97537.1–TolQ-UAY97536.1–TolA-WP_013317028.1–TolB-QWT39951.1), *Rahnella variigena* (TolR-XSE81172.1–TolQ-RKF68651.1–TolA-WP_430318970.1–TolB-RKF68654.1), *Erwinia* sp. (TolR-CAY75014.1–TolQ-CAY75015.1–TolA-WP_023654451.1–TolB-URM09097.1), *Pantoea ananatis* (TolR-ADD76352.1–TolQ-AWQ19528.1–TolA-WP_015700736.1–TolB-BBL31478.1), *Pantoea* sp. (TolR-XLQ81602.1–TolQ-PPS58220.1–TolA-WP_428945588.1–TolB-BBL31478.1), *Escherichia marmotae* (TolR-XKH42590.1–TolQ-PGF74800.1–TolA-WP_428883059.1–TolB-RDS23394.1), *Shigella boydii* (TolR-ABB65284.1–TolQ-ACD09562.1–TolA-WP_000030645.1–TolB-PHU93895.1), *Shigella flexneri* (TolR-KFZ96615.1–TolQ-KFZ96697.1–TolA-WP_000030645.1–TolB-PHU67655.1), *Klebsiella pneumoniae* (TolR-CDO13553.1–TolQ-KFJ72317.1–TolA-WP_429913744.1–TolB-WP_429913744.1), *Klebsiella michiganensis* (TolR-STR40700.1–TolQ-PLL54927.1–TolA-TWW05844.1–TolB-BBW77546.1), *Klebsiella oxytoca* (TolR-XKF40318.1–TolQ-AWF39144.1–TolA-QLX89623.1–TolB-GJK94683.1), *Klebsiella aerogenes* (TolR-KGB07564.1–TolQ-KAE9485139.1–TolA-TWW05844.1–TolB-BBS13227.1), *Lelliottia* sp.(TolR-CAI9412115.1–TolQ-ANG92081.1–TolA-WP_429920331.1–TolB-VDZ88085.1), *Enterobacter hormaechei* (TolR-PTX87772.1–TolQ-PJG38044.1–TolA-WP428887860.1–TolB-CPVU55173.1, *Lelliottia amnigena* (TolR-USR58909.1–TolQ-ANG92081.1–TolA-WP_429920331.1–TolB-VDZ88085.1). (**B**) Phylogenetic analysis of the Pal (peptidoglycan-associated lipoprotein) amino acid sequences. Tree reconstruction and bootstrap validation were performed as described in panel A. The distribution of Pal across diverse genera highlights its evolutionary conservation and fundamental role in outer membrane stabilization. Accessions; *Dickeya* (WP_012770576.1), *Dickeya dadantii* (WP_171860682.1), *Dickeya poaceiphila* (WP_042869557.1), *Enterobacter cloacae* (PDQ13996.1), *Enterobacter hormaechei* (SAA98624.1), *Enterobacter roggenkampii* (RDT58701.1), *Enterobacter* sp. T2 (QIB81364.1), *Erwinia* (WP_013201336.1), *Erwinia aphidicola* (WP_230050002.1), *Erwinia psidii* (WP_124233767.1), *Erwinia tracheiphila* (WP_016191970.1), *Escherichia coli* (KAE9490024.1), *Escherichia coli* str. K-12 substr. MG1655 (NP_415269.1), *Escherichia fergusonii* (PQJ03081.1), *Klebsiella aerogenes* (KGB06706.1), *Klebsiella grimontii* (CAM3928825.1), *Klebsiella michiganensis* (QHO86939.1), *Klebsiella oxytoca* (AVE78362.1), *Klebsiella pneumoniae* (KFJ72966.1), *Klebsiella quasipneumoniae* (VCW95016.1), *Klebsiella variicola* (ACI07041.1), *Mycobacterium* sp. KBS0706 (WP_138893620.1), *Pantoea ananatis* LMG 20103 (ADD76355.1), *Pantoea osteomyelitidis* (WP_397213106.1), *Pantoea* sp. BAV 3049 (WP_158780502.1), *Pantoea* sp. IMH (WP_024967463.1), *Proteus* (WP_012367670.1), *Pseudomonas aeruginosa* (XCM85799.1), *Pseudomonas fluorescens* (KPU57323.1), *Pseudomonas fluvialis* (PKF73415.1), *Pseudomonas lactis* (EIK63724.1), *Pseudomonas lurida* (PRC26985.1), *Pseudomonas sediminis* (PIA71625.1), *Pseudomonas simiae* (AJP54096.1), *Pseudomonas* sp. GOM7 (WAJ36267.1), *Pseudomonas* sp. I3-I5 (UNT14559.1), *Rahnella* (WP_013576426.1), *Salmonella enterica* (TPQ14710.1) and *Yersinia* (WP_005278500.1). (**C**) Structural organization of the Tol–Pal system. TolQ, TolR, and TolA form an inner membrane complex energized by the proton motive force. In the periplasmic space, TolB interacts with the outer membrane-anchored Pal protein, contributing to envelope integrity and membrane cohesion. The conservation of these components reflects their essential structural and physiological roles rather than a specialized or exclusive function in OMV-mediated horizontal gene transfer. Panels A and B were generated using Alignment Explorer/CLUSTALW and MEGA version 11, while Panel C was enhanced by the authors with AI-assisted support (ChatGPT; GPT-4, OpenAI, San Francisco, CA, USA).

The dendrogram derived from the TolA–TolB–TolR–TolQ gene cluster indicated that *Klebsiella* accessions were closer to pathogenic bacteria that cause human diseases, including the genera *Shigella*, *Escherichia*, *Enterobacter* and *Pseudomonas*. *Klebsiella* accessions were shown to be more distantly related to plant pathogens such as *Erwinia* sp., *Pantoea* sp., *P. ananatis* and *Dickeya dadantii* (Figure 6A). The results of bacterial grouping according to the analysis of the PalA protein were similar to those obtained with Tol proteins. *Klebsiella* accessions (*K. michiganensis*, *K. grimontii*, *K. oxytoca*, *K. aerogenes*, *K. quasipneumoniae*, *K. pneumoniae* and *K. variicola*) were closer to *Enterobacter* sp. (*E. cloacae*, *E. hormaechei* and *E. roggenkampii*), *Escherichia coli*, *Escherichia fergusonii* and *Salmonella enterica* (Figure 6B).

This finding reinforces the idea that *K. oxytoca* possesses a highly conserved genetic repertoire for OMV formation, with the potential may contribute to the dissemination of antibiotic resistance genes or virulence factors through HGT. This species has been recognized as an emerging pathogen in nosocomial infections, including cases of antibiotic-associated colitis and sepsis in intensive care units [43]. Therefore, its ability to generate functional vesicles, supported by its phylogenetic proximity to other human pathogens, represents an evolutionary risk factor that deserves attention. The results obtained from the Tol and Pal genes indicate a strong genetic conservation and suggest the idea of using the Tol cluster (TolA–TolB–TolR–TolQ) as molecular markers for the specific detection of *K. oxytoca* and other *Klebsiella* species in genomic surveillance studies, especially in clinical contexts where genetic plasticity facilitates the emergence of multi-resistant strains.

### 2.4. Genomic Characterization of Mobile Genetic Elements, Resistome and Virulome K. oxytoca

Comprehensive genomic analysis of *K. oxytoca* revealed a complex and diverse mobilome, resistome, and virulome, reflecting extensive genomic variability and the presence of multiple mobile genetic elements (MGEs). Functional annotation identified a broad repertoire of antimicrobial resistance (AMR), virulence-associated, and mobility-related genes distributed across the chromosome and multiple genomic islands (GIs). Analysis of genes previously reported in association with OMVs identified a diverse set of AMR-related determinants, primarily corresponding to well-characterized resistance mechanisms. In total, more than 25 AMR genes were detected and classified into four major functional categories: efflux pumps, antibiotic target modification, enzymatic inactivation, and permeability-associated mechanisms (Table S2). A predominance of efflux-related genes belonging to the SMR, RND, MFS, and ABC families was observed, including kpnE, kpnF, qacJ, oqxA, acrA, adeF, kpnG, kpnH, emrR, lptD, and msbA. These genes have been previously associated with resistance to fluoroquinolones, β-lactams, macrolides, aminoglycosides, rifamycins, and disinfectants [44]. Genes involved in antibiotic target modification were also identified, including arnT, eptB, vanG, gyrB, and pbp3, which are associated with structural alterations in lipopolysaccharides or essential cellular targets such as DNA gyrase and penicillin-binding proteins. Enzymatic resistance mechanisms were represented by genes such as fosA5 and OXY-2-2, which encode enzymes capable of antibiotic inactivation through chemical modification. Additionally, genes such as ompA and regulatory elements including marA were detected, both of which are known to influence membrane permeability or porin expression. While several of these genes have been previously reported in OMV-related contexts, their identification here reflects genomic annotation and co-occurrence rather than direct evidence of OMV-mediated HGT.

**Figure 7:**
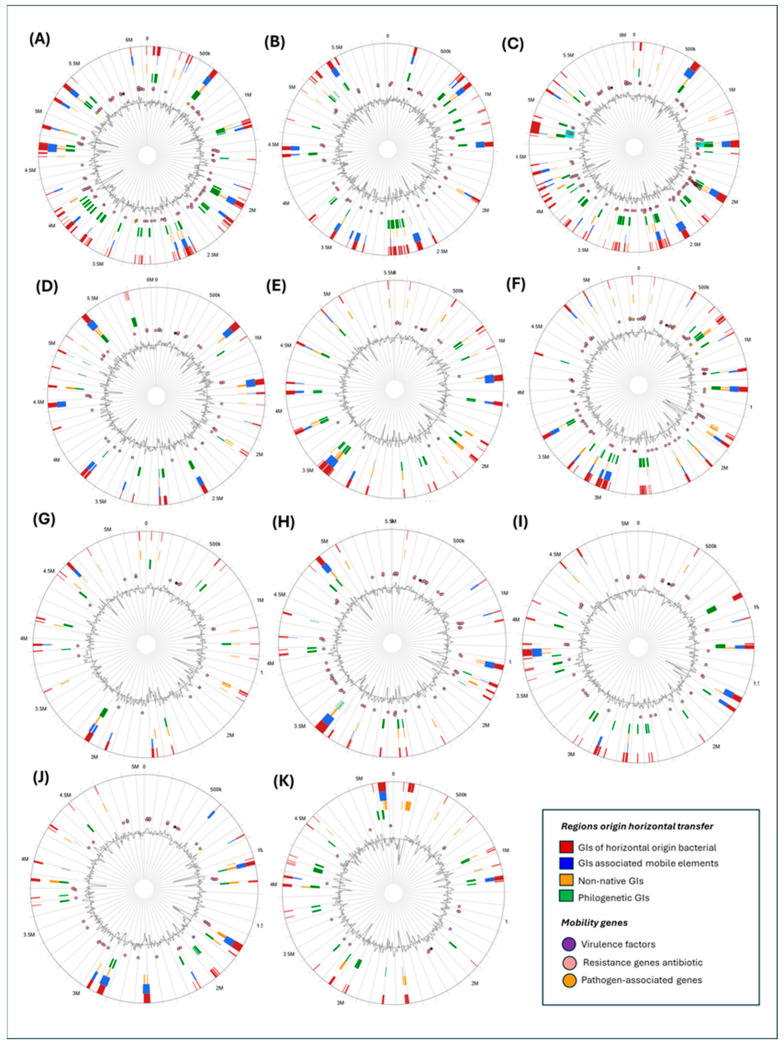
Distribution of horizontally acquired genomic regions and associated functional genes in *Klebsiella* spp. This figure presents circular genome maps illustrating the distribution of genomic regions predicted to be acquired through horizontal gene transfer (HGT) in 11 *Klebsiella* species. Complete genome sequences were analyzed using an integrated approach combining multiple genomic island prediction tools, including IslandPick, IslandPath-DIMOB, and SIGI-HMM. Circular genome maps illustrating predicted genomic islands (GIs) and mobility-related genes in *Klebsiella* species. Predicted genomic islands are indicated according to their inferred origin and characteristics: genomic islands of bacterial origin (red), GIs associated with mobile genetic elements (blue), non-native GIs (green), and GIs exhibiting a phylogenetic signal (orange). The circular tracks also annotate the genomic locations of selected mobility- and host-interaction–related genes, including virulence factors (purple/light purple circles), antibiotic resistance genes (red/pink circles), and pathogen-associated genes (orange/yellow circles). The legend highlights the most prominent features of the figure to facilitate interpretation. Differences in color intensity reflect visualization settings and do not indicate distinct functional categories. The analyzed genomes correspond to the following species: (**A**) *K. oxytoca*, (**B**) *K. grimontii*, (**C**) *K. michiganensis*, (**D**) *K. huaxiensis*, (**E**) *K. quasivariicola*, (**F**) *K. pneumoniae*, (**G**) *K. africana*, (**H**) *K. variicola*, (**I**) *K. quasipneumoniae*, (**J**) *K. aerogenes* and (**K**) *K. indica*. This comparative visualization provides a genome-wide overview of the distribution and co-localization patterns of the mobilome, virulome and resistome within predicted horizontally acquired regions, supporting comparative genomic interpretations related to genome plasticity and adaptive potential in *Klebsiella* spp., without implying direct functional validation.

Comparative analysis of 11 *Klebsiella* genomes revealed the widespread presence of genomic islands acquired through horizontal transfer. GIs were classified based on structural and compositional features, including islands associated with integrases and transposases, regions displaying atypical GC content or codon usage, and islands with irregular phylogenetic distributions. Numerous GIs showed co-localization of resistome, virulome, and pathogen-associated genes, including β-lactamases, efflux pumps, siderophore systems, secretion system components, adhesins, and immune evasion-related factors (Figure 7). Among the species analyzed, *K. oxytoca* (Figure 7A) displayed one of the highest densities of GIs, characterized by an extensive distribution of mobile elements, resistance genes, and virulence determinants. In contrast, species such as *K. grimontii* and *K. africana* (Figure 7B,G) exhibited fewer GIs with more localized genomic distribution and reduced functional diversity. In *K. oxytoca*, resistance genes such as bla_OXY were identified within GIs containing integrases, transposases, and recombination-associated enzymes. Likewise, virulence-associated genes including siderophore systems (entB, iutA), type VI secretion system components, and adhesins were detected at higher frequencies compared to several other species analyzed (Tables S4–S4.3).

Detailed mobilome analysis revealed a high abundance and diversity of MGEs, including class 1 integrases (intI1), transposases from the IS3, IS5, and IS66 families, relaxases, recombinases, and conjugation-related systems such as IncF- and IncHI-type elements (Tables S4–S4.3). Multiple copies of IS5 family transposases were identified, along with genes encoding recombination and replication-associated functions. DNA polymerase I genes were frequently detected within genomic islands and secondary replicon-associated regions, suggesting involvement in the maintenance or replication of mobile DNA [24,45,46]. Numerous hypothetical proteins were found in close proximity to mobile elements (Figure 7), indicating the presence of genomic regions with as yet uncharacterized functions [47]. Key components of conjugative transfer systems, including VirB9, VirB11, and P-type DNA transfer ATPases, were also identified, consistent with the presence of potentially functional conjugative plasmids [48].

Resistome analysis identified a wide array of AMR genes distributed across multiple GIs and mobile regions (Figure 7). In addition to bla_OXY, genes such as aac(6′)-Ib, sul1, qnrS1, and tet(A) were detected, conferring resistance to aminoglycosides, sulfonamides, quinolones, and tetracyclines, respectively (Tables S4–S4.3). These genes have been frequently reported in multidrug-resistant *Klebsiella* clinical isolates [20,49]. Additional resistome-associated elements included genes encoding relaxases, integrases, and reverse transcriptases, commonly associated with conjugative plasmids and mobile regions [20,50]. A broad repertoire of efflux transporters was observed, including members of the ABC, MFS, RND, MacB, and DMT families, along with regulatory genes such as tetR, ramA, lacI, araC, and lysR, which modulate resistance-related gene expression under stress conditions [14,51,52].

Genes conferring resistance to heavy metals, including arsB and arsR, were also identified, consistent with reports of co-selection between metal and antibiotic resistance determinants [53]. Additional genes involved in DNA modification and stress responses were detected, including methylases, type I and III restriction–modification systems, anti-restriction proteins, and SOS response components such as dinI and recA [54].

Virulome analysis revealed a functionally diverse set of determinants associated with iron acquisition, adhesion, secretion systems, biofilm formation, and immune evasion. Siderophore-related genes (entB, fepC, iroE) involved in iron uptake under limiting conditions were identified (Tables S4–S4.3) [55,56]. Adhesion-associated genes such as fimH and mrkD were also detected, encoding fimbrial proteins implicated in attachment to biotic and abiotic surfaces [57,58]. Components of the type VI secretion system (T6SS), including hcp, vgrG, and the ATPase clpV1, were present, along with genes related to type IV secretion systems (T4SS) and interbacterial interaction factors such as rhs [59,60].

Genes involved in capsular polysaccharide biosynthesis and export, including wza and wbaP, were identified, as well as diguanylate cyclases involved in biofilm regulation and lifestyle switching [61,62]. Numerous phage-derived genes encoding capsid, tail, holin, lysin, and baseplate proteins, as well as transcriptional regulators such as AlpA and Cro/CI, were detected, indicating the presence of integrated prophages [63]. Finally, the virulome included genes encoding toxins, peptidases, lysozymes, CbtA, and components of the CidA/LrgAB system, together with fimbrial assembly proteins and Tol–Pal system components involved in cell envelope integrity (Tables S4–S4.3).

This analysis revealed an extensive functional repertoire of efflux transporters, including members of the ABC (ATP-Binding Cassette), MFS (Major Facilitator Superfamily), RND (Resistance–Nodulation–Cell Division), MacB, and DMT (Drug/Metabolite Transporter) families. These efflux systems are widely recognized contributors to multidrug resistance phenotypes through the extrusion of diverse classes of antibiotics and biocides [14]. In addition, several transcriptional regulators associated with antimicrobial resistance and stress responses, such as TetR, RamA, LacI, AraC, and LysR, were identified (Tables S4–S4.3). These regulators are known to modulate the expression of resistance- and virulence-associated genes under environmental or antimicrobial stress conditions [51,52].

The resistome of *K. oxytoca* also includes genes conferring tolerance to heavy metals, such as arsB and arsR, associated with arsenic resistance (Tables S4–S4.3). The co-occurrence of metal resistance and antibiotic resistance determinants has been previously reported and may contribute to the persistence of resistance traits under selective environmental pressures [53]. Furthermore, genes involved in DNA modification and stress response, including DNA methylases, type I and III restriction endonucleases, anti-restriction proteins, and components of the SOS response such as dinI and recA, were detected, indicating genomic features associated with adaptation to antibiotics, phage exposure, and other external stressors [54].

Genomic analysis also demonstrated a complex and functionally diverse virulome, comprising multiple determinants related to iron acquisition, adhesion, secretion systems, biofilm-associated traits, and immune interaction, all of which are commonly associated with pathogenic potential in both clinical and environmental contexts. Among the virulence-associated genes identified were siderophore-related genes such as entB, fepC, and iroE (Tables S4–S4.3), which are involved in iron uptake under iron-limited conditions [55,56]. In addition, adhesion-associated genes including fimH and mrkD were detected, encoding fimbrial components implicated in attachment to biotic and abiotic surfaces and biofilm formation [57,58].

Components of the type VI secretion system (T6SS), such as hcp, vgrG, and the ATPase clpV1, were identified, consistent with systems involved in interbacterial interactions and competitive fitness [59]. Genes related to type IV secretion systems (T4SS), including rhs, were also detected and are typically associated with conjugative or contact-dependent interactions between bacterial cells [60]. Additionally, genes involved in capsular polysaccharide biosynthesis and export, such as wza and wbaP, were identified, reflecting conserved mechanisms linked to surface structure and protection against host immune defenses [61]. Genes encoding diguanylate cyclases, which regulate intracellular cyclic di-GMP levels and influence biofilm formation and motility, were also present [62].

The genome further contained numerous phage-related genes, including those encoding capsid proteins, tail proteins, holins, lysins, baseplate assembly components, and phage transcriptional regulators such as AlpA and Cro/CI. These elements indicate the presence of integrated prophages, which are commonly associated with genomic diversification and horizontal gene transfer via specialized transduction [63]. Finally, the virulome includes genes encoding bacterial toxins (e.g., cbtA), enterotoxins, peptidases (S14, P60), lysozymes, and components of the CidA/LrgAB systems, which are involved in cell lysis regulation and bacterial persistence. Fimbrial assembly proteins, fimbrial chaperones, and Tol–Pal system components were also detected, reflecting their conserved roles in cell envelope integrity and envelope-associated processes rather than direct evidence of OMV-mediated gene transfer (Tables S4–S4.3).

## 3. Discussion

### 3.1. Evolution and Genomic Dynamics of the Klebsiella Genus

Analysis of the *Klebsiella* spp. pangenome revealed a highly conserved genomic structure with sufficient variability to support an open pangenome model. This genomic dynamic allows these bacteria to rapidly adapt to new environments, particularly those characterized by high selective pressure, such as hospitals [17]. The predominance of the core genome (~92%) indicates strong evolutionary conservation, suggesting that basic cellular functions are highly conserved among the analyzed species. However, the presence of 8% accessory and unique genes demonstrates a genetic pool that enhances phenotypic variability, differential pathogenicity, and multidrug resistance to antibiotics [18].

One of the most relevant aspects of this study is the heterogeneity in the distribution of homologous families among *Klebsiella* species, observed especially in *K. pneumoniae, K. quasipneumoniae* and *K. variicola* (phylogenetically related species) compared to more divergent species (*K. indica*). This pattern suggests that mobile genetic elements (MGEs) (plasmids, integrons, transposons) have played a key role in shaping species-specific gene content. The intensified gene flow between *K. michiganensis, K. oxytoca* and *K. grimontii* (with more than 4400 shared families) reinforces the hypothesis of frequent horizontal transfer, possibly facilitated by cohabitation in clinical settings and by MGE-driven genomic plasticity. This genomic plasticity is a key feature of open pangenomes, especially in bacterial genera associated with nosocomial infections [24].

The results of the comparative analysis of homologous families among *Klebsiella* spp. genomes offer insight into the evolutionary relationships and genomic conservation within the genus. The marked differences in species homology levels suggest that *Klebsiella* constitutes a highly diverse group, with lineages that have evolved at distinct rates, likely influenced by their hosts and specific selective pressures. The low number of homologous families shared by *K. indica* with the rest of the species suggests a specialization process that has led to the loss or acquisition of specific genes. This divergence reflects a lower capacity for horizontal genetic exchange with other species in the genus. In contrast, the high homology between *K. grimontii* and *K. oxytoca* supports their grouping within the *K. oxytoca* complex and suggests that they share a functional set of genes that could be related to their pathogenicity in humans. This finding highlights the need for close genomic surveillance, given that species in this complex have been linked to nosocomial infections and extended-spectrum β-lactamases (ESBL) production.

The genomic similarity between *K. pneumoniae* and *K. quasipneumoniae*, as well as between *K. quasivariicola* and *K. variicola*, could facilitate the horizontal transfer of resistance and virulence genes through mobile elements, such as plasmids and outer membrane vesicles, strengthening the genomic plasticity of the group. The analysis of homologous families not only reaffirms previously described evolutionary relationships but also raises new questions about the genomic and adaptive dynamics within the *Klebsiella* genus, especially in a clinical context where antimicrobial resistance is a growing threat.

Although classical mechanisms such as conjugation, transduction, and transformation have been widely documented, outer membrane vesicles (OMVs) as emerging vectors in gene transfer could represent an alternative transfer mechanism for the transport of resistance genes, virulence factors, and other adaptive elements. In Gram-negative bacteria, OMVs have demonstrated the ability to encapsulate and transfer extrachromosomal DNA, which would confer them an active role in the evolution of bacterial populations under antibiotic pressure [64,65]. Although this mechanism has been described mainly in *Escherichia coli* and *Pseudomonas aeruginosa*, its relevance in *K. oxytoca* is still poorly characterized. The identification of genes involved in OMV biogenesis in clinical strains of *K. oxytoca* could explain, at least in part, their ability to efficiently disseminate resistance genes in hospital settings. This OMV-facilitated transfer could act in synergy with EGMs, generating a highly efficient system for the acquisition and diffusion of adaptive determinants [42]. Furthermore, the unique genes identified in the pangenome could include determinants involved in this emerging transfer pathway.

The pan-genome results highlight the high genomic plasticity of *Klebsiella*, which allows it to adapt to clinical environments through the acquisition of resistance genes. Although the observed gene distribution is compatible with features commonly associated with an open pangenome, the limited sampling and absence of quantitative growth-curve modeling (e.g., Heaps’ law fitting) preclude definitive conclusions regarding pangenome openness. Taken together, the pangenome analysis presented here should be regarded as a descriptive and comparative framework based on reference genomes, rather than as a comprehensive representation of genus-wide genomic diversity. Expanded sampling, including multiple strains per species and quantitative modeling of pangenome growth, will be required to robustly characterize core and accessory genome dynamics in *Klebsiella*.

The presence of genes such as blaKPC-2 and blaCTX-M-15 in strains highlights their role in the dissemination of resistance to broad-spectrum beta-lactam antibiotics [24,42]. The detection of genes such as oqxA and oqxB, which are part of a RND (resistance-nodulation-division) efflux system and have been previously identified in extracellular vesicles, suggests a possible indirect defense mechanism through the sequestration or expulsion of antibiotics into the environment [66,67]. Furthermore, the presence of genes such as tolC, involved in both the assembly of efflux systems and the release of OMVs, establishes a structural and functional link between antimicrobial resistance and vesicular secretion. These vesicles can act as vehicles for horizontal gene transfer, carrying DNA, RNA, and proteins between bacteria, contributing to the rapid dissemination of resistance in microbial communities [36,68]. Taken together, these results support the hypothesis that OMVs may represent a complementary and still incompletely characterized pathway that could operate alongside classical horizontal gene transfer mechanisms, such as conjugation, transduction, and transformation. In the context of this genome-based study, OMV involvement should be considered hypothetical and requires functional validation.

**Figure 8:**
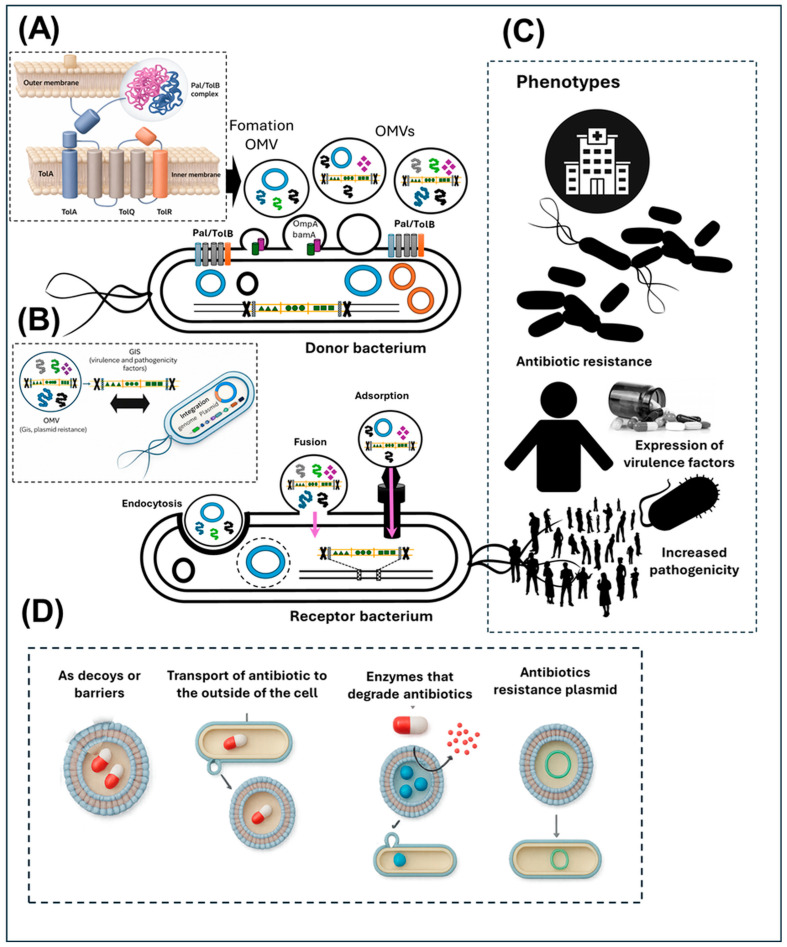
Conceptual model of OMV mediated horizontal gene transfer in *Klebsiella oxytoca.* This figure presents a representative and integrative conceptual model illustrating potential mechanisms by which OMVs may contribute to HGT in *K. oxytoca*. The model synthesizes current knowledge and genomic evidence discussed in this study and does not represent direct experimental validation. (**A**) OMV biogenesis and genetic cargo packaging in the donor bacterium. The upper left panel depicts donor proteins (e.g., OmpA and BamA), which are indicated to illustrate their proposed roles in vesicle formation and release. OMVs of varying sizes are shown budding from the outer membrane and carrying heterogeneous cargo, including plasmids and chromosomal fragments containing genomic islands (GIs). These regions may encode functions associated with antibiotic resistance, virulence, and host interaction, as represented schematically. (**B**) Interaction with recipient cells and genetic uptake. The lower left and central panels illustrate potential modes of interaction between OMVs and recipient bacteria, including membrane association, fusion, or uptake-like processes. Upon interaction, vesicle-associated content may be released into the periplasmic or cytoplasmic space. The schematic highlights possible fates of transferred genetic material, including maintenance as plasmids or integration into the chromosome through recombination-related mechanisms. Integrases and transposases are depicted to indicate enzymatic activities commonly associated with the mobilization and stabilization of genomic islands. (**C**) Functional consequences at the cellular and population level. The right panel summarizes potential phenotypic outcomes following OMV-mediated gene acquisition, including altered antibiotic susceptibility profiles, expression of virulence-associated traits, and increased adaptive or pathogenic potential. These outcomes are presented as theoretical and associative consequences derived from horizontal gene transfer events and are not intended to imply direct causality. (**D**) Proposed contributions of OMVs to antibiotic resistance dynamics. The lower panel illustrates several mechanisms by which OMVs may contribute to antimicrobial resistance, including: (i) sequestration of antibiotics acting as decoys or physical barriers; (ii) dissemination of resistance-associated plasmids; (iii) contribution to antibiotic efflux-related processes; and (iv) transport of antibiotic-inactivating enzymes, such as β-lactamases. Collectively, these mechanisms highlight the potential role of OMVs in bacterial survival under antimicrobial pressure. The figure is original and was created by the authors. Panel D was remastered by the authors using ChatGPT-4, (OpenAI) as an AI-assisted tool.

Genes are involved in vesicle formation and their role in the horizontal transfer of genetic material in *K. oxytoca*. The formation of outer membrane vesicles (OMVs) in Gram-negative bacteria such as *K. oxytoca* is a multifactorial process that responds to both structural alterations in the cell envelope and environmental stimuli (Figure 8). Several studies have shown that the disruption of the physical links between the outer membrane and peptidoglycan, mediated by proteins such as OmpA, Pal and Lpp, promotes spontaneous vesiculation by weakening the structural cohesion of the envelope. In *Escherichia coli*, the deletion of ompA and lpp can increase OMV production up to sixfold, and a similar pattern is presumed to occur in *K. oxytoca* [32,69].

The BAM (Beta-barrel Assembly Machinery) complex, composed of the subunits bamA–E, is essential for the assembly of β-barrel proteins at the outer membrane. Mutations in bamA and bamB can cause accumulation of misfolded precursors, inducing compensatory hypervesiculation to relieve cellular stress [70]. This phenomenon is amplified by the inactivation of periplasmic chaperones such as SurA and proteases such as DegP, whose function is to assist in the folding and degradation of aberrant proteins in the periplasmic space [71,72]. Furthermore, the Tol–Pal system, which physically connects the outer membrane to the peptidoglycan, plays a key role in maintaining structural integrity. Disruption of genes such as tolA, tolB, pal and tolR has been linked to increased OMV release, attributed to a loss of mechanical tension between the layers of the cell envelope [36,73]. This weakening allows spontaneous protrusion of vesicles enriched in lipopolysaccharide (LPS) and virulence-associated proteins.

Outer membrane lipid homeostasis regulates vesiculation. Transport proteins such as MlaA and MsbA ensure the balance of phospholipids and LPS. Mutations in these systems can induce irregular lipid accumulations, generating highly curved zones that favor OMV formation [63,69]. This alteration not only increases the frequency of vesicle release but also modifies their contents, making them more suitable for transporting virulence factors or genetic material. Under stress conditions, the two-component systems PmrA/B and PhoP/Q play an essential role in regulating vesiculation. These environmental sensors respond to stimuli such as the presence of antimicrobial peptides or low levels of Mg^2+^, inducing membrane remodeling that facilitates OMV release as a defense strategy and as a means of eliminating toxic compounds [74,75].

On the other hand, LPS and lipid A biosynthesis influence vesicular dynamics. Genes of the lpx cluster (e.g., lpxA, lpxC, lpxD, lpxM) are involved in the acylation of lipid A, a process that affects membrane asymmetry. Alterations in this pathway increase membrane fluidity and curvature, favoring vesiculation [36,75]. Additionally, the gmhA and gmhD genes, involved in the synthesis of the LPS core oligosaccharide, are essential for its anchoring; mutations in these genes result in the formation of incomplete structures, which favor vesicular extrusion as a removal mechanism [69]. Peptidoglycan synthesis, regulated by genes such as murA-F and mraY, represents another critical point. Its disruption generates structural tensions that can be relieved by the release of OMVs [32]. Similarly, genes such as gpsA, plsB and plsC, involved in phospholipid synthesis, can modulate the lipid composition of OMVs, affecting their ability to package and transport metabolites or nucleic acids [76]. The selD gene, involved in selenophosphate biosynthesis, appears to play an indirect role in vesiculation under oxidative stress conditions. selD has been proposed to be involved in cellular detoxification pathways in which OMVs act as protective elements, removing damaged proteins or reactive species [42].

Taken together, the genes identified in *K. oxytoca* reflect a conserved vesiculogenic genetic architecture in enterobacteria, which responds to multiple structural and environmental stimuli. These genes are primarily involved in envelope integrity, stress response, and vesiculation dynamics. While they may contribute to conditions permissive for vesicle-associated transport, no functional transfer of resistance genes via OMVs can be inferred from genomic data alone [77]. Plasmids, integrative conjugative elements (ICEs), integrons, and bacteriophages remain the most extensively validated drivers of horizontal gene transfer in *Klebsiella* spp. The genomic patterns observed here are fully compatible with these mechanisms, and the relative contribution of OMVs cannot be resolved without direct experimental evidence. Therefore, OMV-associated hypotheses should be viewed as complementary rather than substitutive. Thus, the involvement of OMVs proposed in this work should be regarded as a recently recognized evolutionary possibility, supported by comparative genomic patterns, and not as a newly emerged mechanism in contemporary bacterial populations.

### 3.2. Evolutionary Implications of Proteins Associated with Outer-Membrane-Vesicle-Mediated Horizontal Gene Transfer in Klebsiella spp.

The phylogenetic analyses performed in this study provide robust evidence for the conservation and concerted evolution of genes associated with outer membrane structure and vesicle-related processes in *Klebsiella* spp. Although OMVs have been proposed as potential auxiliary vehicles for genetic material in certain bacterial systems, complementing classical HGT mechanisms such as conjugation, transformation, and transduction [42,77], their contribution to gene transfer remains context-dependent and experimentally limited. Accordingly, in this work, OMV-associated processes are considered within a genome-based inferential framework rather than as demonstrated mechanisms of functional gene transfer. In clinical settings, OMVs have been associated with the dissemination of antimicrobial resistance determinants and virulence-related factors under specific experimental conditions; however, their relative contribution compared to established HGT pathways remains uncertain (Figure 8).

It is therefore essential to conceptually distinguish between different categories of genes frequently discussed in relation to OMVs. A substantial proportion of OMV-associated genes—including components of the Tol–Pal system, the β-barrel assembly machinery (BAM), and other envelope structural proteins—are essential for cell envelope integrity and are broadly conserved across Gram-negative bacteria. The phylogenetic conservation of these genes primarily reflects strong evolutionary constraints related to membrane stability, cell division, and envelope homeostasis, rather than specialization for vesicle-mediated gene transfer. A second group of genes has been reported to modulate vesiculation rates indirectly, typically as a consequence of envelope stress, misfolded protein accumulation, or regulatory responses. In contrast, only a very limited number of mechanisms have been experimentally shown to mediate selective DNA cargo packaging into OMVs, and such evidence remains scarce, organism-specific, and poorly conserved. Accordingly, the genes analyzed in the present phylogenetic framework predominantly belong to the first category.

Phylogenetic reconstructions revealed clear intraspecific relationships within the *Klebsiella* clade, consistent with stabilizing selection acting on genes such as ompA, bamA, pal, and degP. These genes are conserved across species of the genus, indicating selective pressure to maintain envelope-associated functions that may indirectly influence vesiculation as a byproduct of structural integrity and stress responses [32,69]. The presence of these genes in both commensal and pathogenic species supports their fundamental physiological role rather than a lineage-specific adaptation for OMV-mediated HGT. Moreover, their conservation across isolates from diverse ecological and clinical contexts suggests that their persistence is driven primarily by essential cellular functions rather than selective optimization for gene transfer (Figure 8). Phylogenetic analyses across multiple taxonomic levels further indicate that these systems likely originated from a common ancestor within the Gammaproteobacteria and diversified in response to ecological pressures while retaining core structural functionality [78,79].

One of the most conserved envelope-associated systems identified in this study is the Tol–Pal system, which comprises the proteins TolQ, TolR, TolA, TolB, and Pal. This multiprotein complex plays a central role in maintaining outer membrane integrity, coordinating cell division, and supporting envelope stability in Gram-negative bacteria [80,81]. Although the phylogenetic analyses of Tol–Pal components are technically robust and display the expected clustering within Enterobacterales, their evolutionary interpretation requires caution. The high degree of conservation observed for Tol–Pal proteins is best explained by strong evolutionary constraints associated with envelope architecture and cellular viability, rather than by adaptive specialization for OMV-mediated HGT. Previous studies have shown that tol/pal genes are organized into conserved operons (e.g., ybgC–tolQ–tolR–tolA and tolB–pal–cpoB), although alternative transcriptional arrangements have also been reported [82,83,84]. Functional disruptions of this system have been linked to compromised membrane stability, reduced virulence, and increased antimicrobial susceptibility across several pathogenic species, including *Escherichia coli*, *Salmonella enterica*, *Shigella flexneri*, *Pseudomonas aeruginosa*, and *Klebsiella pneumoniae* [85,86,87,88]. These observations reinforce the interpretation of Tol–Pal as a broadly conserved envelope-maintenance system rather than a specific driver of vesicle-mediated gene transfer.

From an applied perspective, OMVs remain of considerable biotechnological interest, particularly for vaccine development, due to their immunogenic properties without direct pathogenicity. Successful examples include OMV-based vaccines against Neisseria meningitidis serogroup B [89,90] and ongoing efforts targeting *Shigella flexneri* [91]. Within this context, Pal has been evaluated as a vaccine antigen in recombinant, peptide-based, and plasmid DNA formulations, demonstrating protective effects in animal models [92]. However, while sequence variation in Tol–Pal components may reflect lineage-specific divergence, the present analysis does not support their use as molecular markers for *K. oxytoca* or as indicators of OMV-mediated gene transfer without dedicated specificity and functional validation. Overall, the phylogenetic conservation observed in this study is most appropriately interpreted as evidence of structural and functional constraints acting on essential envelope-associated systems. While OMV production may occur as a secondary consequence of envelope perturbations or stress responses, the available genomic and phylogenetic data support a complementary, context-dependent role for OMVs rather than specialization for horizontal gene transfer. This interpretation aligns with current experimental evidence and underscores the need for functional validation to elucidate the precise contribution of OMVs to microbial evolution and antimicrobial resistance dissemination.

### 3.3. Genomic Plasticity, Dynamic Mobilome, Functional Resistome and Complex Virulome in the K. oxytoca Model

The present genomic analysis highlights *K. oxytoca* as one of the members of the *Klebsiella* genus exhibiting pronounced genomic plasticity, reflected in the abundance and diversity of genes acquired through HGT. This plasticity is manifested in a high density of genomic islands (GIs) harboring functional determinants associated with antimicrobial resistance and virulence. When compared with other species of the genus, such as *K. aerogenes*, *K. indica*, or *K. huaxiensis*, which display fewer GIs and a lower density of pathogenicity-related genes, *K. oxytoca* emerges as a relevant reservoir of clinically and epidemiologically significant genetic traits [20,93].

Historically, *K. pneumoniae* has received the greatest attention within the *Klebsiella* complex due to its prevalence in nosocomial infections and its multidrug-resistant phenotypes. However, the present results indicate that *K. oxytoca* harbors an equally complex—and in some cases more diverse—repertoire of mobile genetic elements, including integrases, transposases, and conjugative plasmid-associated components [94,95]. The frequent co-localization of these mobile elements with resistome and virulome genes suggests that *K. oxytoca* represents a recombination-prone genomic background, a feature widely reported across *Klebsiella* spp. and other Enterobacterales. Importantly, such genomic architectures are primarily associated with well-established HGT mechanisms, including conjugation, transposition, and phage-mediated transduction. The presence of resistance and virulence genes within GIs should therefore be interpreted as a hallmark of classical horizontal gene transfer rather than as evidence for their transport via outer membrane vesicles (OMVs) [25].

From an evolutionary perspective, the observed genomic patterns are consistent with sustained selective pressures favoring the retention and diversification of functionally relevant GIs. These pressures are likely linked to antibiotic exposure, host-associated environments, and ecological competition, rather than to specialization toward a single gene transfer pathway. This interpretation underscores the importance of including *K. oxytoca* in molecular surveillance initiatives and supports the need for future functional studies addressing gene expression dynamics, colonization capacity, resistance phenotypes, and environmental adaptability [96].

The recurrent co-localization of resistance genes—such as bla_OXY, fosA, and oqxA—with virulence determinants within mobile regions is of particular concern, as it facilitates their co-transfer to other bacterial strains through mechanisms such as conjugation or specialized transduction [24,47]. In this context, the identification of multiple conjugative systems, including IncF- and IncHI-type plasmid-associated proteins, relaxases, and VirB/T4SS components, strongly supports classical conjugation as a dominant driver of gene dissemination in *K. oxytoca*. These mechanisms likely account for a substantial proportion of the mobilome dynamics observed in this species. Any potential involvement of OMV-associated processes, if present, should therefore be regarded as auxiliary and context-dependent, requiring direct experimental validation before functional relevance can be inferred.

Consistent with its genomic profile, *K. oxytoca* has been increasingly reported in nosocomial infections, particularly among immunocompromised patients or individuals with implanted medical devices, highlighting its clinical relevance in high-risk settings [2,97]. The detection of siderophore systems and type VI secretion system (T6SS) components, which are involved in iron acquisition, interbacterial competition, and immune evasion, further supports its classification as an opportunistic pathogen with the capacity for persistence and dissemination within the human host [59,93]. Although *K. oxytoca* remains less frequently reported than *K. pneumoniae* in traditional surveillance frameworks, its genomic complexity, diversity of mobile elements, and ability to integrate clinically relevant genes justify its prioritized inclusion in comparative pathogenomics studies within the *Klebsiella* genus.

At a broader scale, the mobilome of *K. oxytoca* is characterized by a diverse assemblage of mobile genetic elements, including transposases (notably from the IS5 and IS110 families), integrases, recombinases, relaxases, and conjugative ATPases. This flexible genomic architecture enables the acquisition, reorganization, and dissemination of adaptive traits associated with antimicrobial resistance, virulence, and environmental persistence [23,24]. The abundance of mobile DNA polymerases embedded within insertion regions and GIs further reflects extensive genomic remodeling under selective pressures such as intensive antimicrobial exposure [98]. In addition, the detection of structural and regulatory phage-related genes, including AlpA and Cro/CI, indicates the presence of integrated prophages capable of mediating gene transfer via specialized transduction, a well-documented mechanism in bacterial evolution [63,99].

The identification of toxin–antitoxin systems, such as SymE and VapC, suggests a role in plasmid maintenance, population control, and stress responses, contributing indirectly to the stability of mobile elements within the resistome [100,101]. Moreover, genes encoding components of type VI and type IV secretion systems (T6SS and T4SS), as well as capsular polysaccharide export machinery, highlight traits associated with interspecific competition, immune evasion, and biofilm formation, collectively enhancing the pathogenic potential of the analyzed strain [100,101]. The coexistence of mobile elements, resistance determinants, and virulence factors thus reflects an evolutionary convergence commonly observed in multidrug-resistant opportunistic pathogens.

The resistome further includes genes encoding ABC- and MFS-type efflux pumps, along with determinants conferring resistance to heavy metals such as arsenic and arsenate. These systems contribute to antimicrobial tolerance and environmental detoxification and may be co-selected under stress conditions [102,103]. Their frequent association with transcriptional regulators suggests inducible expression in response to environmental cues, enabling rapid adaptive responses. The virulome encompasses genes encoding fimbriae, toxins, peptidases, and secretion systems that support adhesion, colonization, and invasion. Additionally, components of the Lrg/Cid system, involved in autolysis regulation and extracellular DNA release, were detected, linking biofilm formation with horizontal gene acquisition [101]. The presence of multiple DNA methylation systems, restriction–modification modules, and anti-restriction proteins further illustrates the regulatory balance between protection against foreign DNA and the selective retention of beneficial mobile elements [104,105].

Finally, the detection of genes associated with specialized metabolic functions, including nitrogen fixation, suggests that HGT has contributed not only to pathogenicity and resistance but also to broader ecological versatility [106]. Collectively, these findings support the conclusion that classical horizontal gene transfer mechanisms—particularly conjugation and phage-mediated transduction—are the primary evolutionary forces shaping the mobilome, resistome, and virulome of *K. oxytoca*. While OMV-associated processes have been proposed in other bacterial systems, the genome-based evidence presented here does not support their interpretation as a distinct or independent evolutionary pathway in this species. Instead, OMVs are more appropriately considered a complementary and context-dependent phenomenon whose functional role remains to be demonstrated experimentally.

### 3.4. Evolutionary Synergy in Antimicrobial Resistance in K. oxytoca

Comparative genomics highlight the fundamental role of outer membrane vesicles (OMVs) as vehicles of evolutionary adaptation in *Klebsiella* spp., especially under antimicrobial pressure. The high presence of active efflux systems of toxic compounds, such as the RND (Resistance–Nodulation–Division) and MFS (Major Facilitator Superfamily) efflux pumps, highlights their participation in resistance mechanisms. In particular, systems such as AcrAB-TolC, oqxA and kpnG/kpnH have been identified in OMVs, reinforcing their role as a primary defense strategy against antibiotics [6,36].

The involvement of these genes in OMV-mediated horizontal transfer suggests a complementary evolutionary dynamic acquired by certain *Klebsiella* species, allowing them to respond effectively to selective pressures in clinical settings. OMVs not only carry functional proteins but also DNA and RNA fragments that can be incorporated by recipient bacteria, effectively conferring novel phenotypic resistance capabilities [107,108]. In this context, regulatory genes such as marA (multiple antibiotic resistance activator) and CRP (cyclic AMP receptor protein) could play an indirect role by modulating the expression of various antimicrobial resistance (AMR) genes in recipient cells. This transcriptional regulation capacity favors the activation of defense pathways against environmental or pharmacological stimuli1 [21]. Furthermore, the detection of genes involved in lipopolysaccharide (LPS) modifications, such as arnT and eptB, in the content of OMVs, suggests that these elements could play a dual role: strengthening resistance in the transmitting cell and conferring protective structural characteristics to recipient bacteria, especially against antibiotics such as colistin, which act on the outer membrane [78] (Figure 8).

Although formal enrichment, frequency-based, or normalization analyses were not performed, the comparative genomic framework presented here highlights the presence, organization, and conservation of OMV-related genes in *K. oxytoca*. This qualitative approach provides functional context for genome annotation while avoiding overinterpretation. Future studies incorporating quantitative enrichment and normalization strategies across closely related species may further refine species-specific patterns.

Another relevant finding is the presence of genes encoding antibiotic inactivation enzymes, such as OXY-2-2 (an OXY-type β-lactamase specific to *K. oxytoca*) and fosA5 (a fosfomycin-modifying enzyme). Their inclusion in OMVs supports the hypothesis of a “collective resistance” mechanism, whereby bacteria release enzymes into the environment that inactivate antibiotics before they enter cells. This phenomenon, already documented in microbial consortia, represents an important therapeutic barrier in nosocomial infections [107]. Furthermore, although certain genes commonly associated with resistance, such as vanG (vancomycin resistance), PBP3 (penicillin-binding protein 3), and gyrB (DNA gyrase subunit B), are not usually found in OMVs, their transfer through other mobile genetic elements, such as plasmids, highlights the coexistence of multiple routes of genetic dissemination in *Klebsiella* [108,109].

In this study, genomic analysis of *K. oxytoca* supports the notion that outer membrane vesicles (OMVs) are not merely inert subcellular structures, but dynamic entities with an active role in horizontal gene transfer, maintenance of the bacterial resistome, and the collective dissemination of resistance determinants (Figure 8). This biological functionality of OMVs contributes significantly to the evolutionary complexity of *Klebsiella* spp. as opportunistic pathogens and justifies their consideration in advanced genomic surveillance strategies, as well as in the design of novel antimicrobial therapies.

### 3.5. Alternative Explanations for Horizontal Gene Transfer: Plasmids, ICEs and Prophages

Although this study explores the evolutionary plausibility of OMVs as auxiliary contributors to genetic dissemination in *K. oxytoca*, the genomic evidence strongly indicates that classical mobile genetic elements constitute the primary drivers of HGT in this species. Plasmids, integrative and conjugative elements (ICEs), and prophages are well-established, experimentally validated mechanisms that collectively provide a parsimonious and comprehensive explanation for the observed patterns of genome plasticity, resistome expansion, and virulome diversification in *Klebsiella* spp. and other Enterobacterales [20,25,63,98].

Mobilome analysis revealed multiple plasmid-associated signatures, including relaxases, type IV secretion system (T4SS) components (e.g., VirB proteins), replication and partitioning functions, and Inc-type conjugative backbones. These features are fully consistent with active plasmid-mediated conjugation, which is widely recognized as the dominant route for the dissemination of antimicrobial resistance genes in *Klebsiella*, particularly in clinical and hospital-associated lineages [20,25]. The frequent co-localization of resistance determinants such as bla, qnr, aac, tet, and sul genes with plasmid-related regions further supports plasmids as the principal vehicles shaping the resistome architecture [24,25].

Beyond plasmids, integrative and conjugative elements and genomic islands play a central role in chromosomal diversification and long-term genome evolution. Numerous genomic islands identified in *K. oxytoca* contained integrases, recombinases, and transposases characteristic of ICEs and composite transposons. These elements enable chromosomal integration, excision, and conjugative transfer, facilitating both stable inheritance and horizontal dissemination of adaptive traits [23,24,63,98]. In *Klebsiella* species, ICE-associated regions are strongly linked to clusters of resistance, virulence, and stress response genes, providing a robust explanation for their structured genomic distribution without invoking unconventional transfer mechanisms [37,46,63].

Prophage-related regions were also abundant in the genome, encoding capsid, tail, holin, lysin, and regulatory proteins such as Cro/CI and AlpA, indicative of functional or remnant bacteriophages. Phage-mediated transduction represents a classical and well-documented mechanism of horizontal gene transfer and lysogenic conversion, contributing to the acquisition of virulence factors, regulatory modules, and fitness-associated genes [99,103]. The coexistence of prophage elements with resistance and virulence loci suggests that specialized or generalized transduction has likely contributed to the observed genomic architecture of *K. oxytoca*, in agreement with previous studies on Enterobacterales genome evolution [63,99,103].

In contrast, while OMVs have been experimentally shown in selected bacterial models to encapsulate DNA and resistance determinants [26,27,64,65], genomic data alone cannot demonstrate active OMV-mediated gene transfer. OMV biogenesis is primarily associated with envelope stress responses, membrane remodeling, immune modulation, and intercellular communication rather than with selective cargo packaging or targeted genetic exchange [32,36,72,77]. Therefore, the mere presence of OMV-associated genes in genomic datasets should not be interpreted as direct evidence of functional HGT via vesicles [26,32,36].

Notably, the OMV-associated genes identified in this study largely correspond to conserved envelope maintenance systems, particularly components of the Tol–Pal complex. These systems play essential roles in outer membrane integrity, lipid homeostasis, and cell envelope stabilization, and are widely conserved across Gram-negative bacteria [73,80,81,82,83,84,85,86,87,88,89,90,91,92,93,94]. Although Tol–Pal dysfunction can influence vesiculation rates, these proteins are not specialized for DNA loading or delivery, reinforcing the view that OMVs function primarily as stress-adaptive or immunomodulatory structures rather than autonomous vectors of horizontal gene transfer [36,77,80].

Taken together, the resistome, virulome, and mobilome profiles observed in *K. oxytoca* are best explained by the combined and well-characterized action of plasmids, ICEs, and prophages as the dominant evolutionary forces driving genome plasticity and antimicrobial resistance dissemination [20,35,63,98]. Within this established framework, OMVs should be regarded as a complementary, context-dependent, and hypothesis-generating mechanism whose contribution to horizontal gene transfer in *Klebsiella* remains speculative and requires direct experimental validation [6,7,26,77,106].

## 4. Materials and Methods

### 4.1. Genome Study and Analysis of OMV-Associated Genes in Klebsiella

In this study, the genomes of 11 species of the genus *Klebsiella* were used: *Klebsiella oxytoca* (GCF_001022115.1), *Klebsiella africana* (GCA_016804125.1), *Klebsiella guasivariicola* (GCF_002269255.1), *Klebsiella quasipneumoniae* (GCF_001278905.1), *Klebsiella variicola* (GCF_000828055.2), *Klebsiella michiganensis* (GCF_000783895.2), *Klebsiella pneumoniae* (GCA_000764615.1), *Klebsiella huaxiensis* (GCF_003261575.2), *Klebsiella grimontii* (GCF_013590775.1), *Klebsiella indica* (GCF_005860775.1) and *Klebsiella aerogenes* (GCF_000755545.1). The *Klebsiella* genome was obtained from the NCBI (National Center for Biotechnology Information) Genome database, URL: https://www.ncbi.nlm.nih.gov/ (accessed on 25 February 2025). The sequence set was generated with DRAM and annotations were made with DomainAnnotation v1.0, which identifies protein domains from domain libraries. To do this, the *K. oxytoca* reference genome was compared to all domain libraries in the Department of Energy Systems Biology Knowledge Base platform, URL: https://www.kbase.us/ (accessed on 18 March 2025). The domain libraries in KBase include: A) the NCBI Conserved Domain Database (CDD) and the Groups of Orthologous Groups (COG); B) NCBI CDD models from the NCBI Conserved Domain Database (CDD), version 3.16; C) SMART (Simple Modular Architecture Research Tool) version 6.0, from CDD; D) PRK (Protein Clusters version 6.0, from CDD; E) Pfam version 31.0 hidden Markov models (HMMs); and F) TIGRFAMs version 15.0 HMM from the J. Craig Venter Institute. RPS-BLAST version 2.2.31 from the BLAST+ package at NCBI. For all three HMM libraries (Pfam, TIGRFAMs, and NCBIfam), KBase runs HMMER version 3.1b2, which identifies all domain hits as at least as significant as the family-specific boundary confidence identified by the curators of each model.

In the *K. oxytoca* genome, genes involved in vesicle biogenesis, outer membrane remodeling and OMV release were identified using the software for rapid annotation of prokaryotic genomes ProkkaAnnotation v3.2.117. To identify functional pathways of outer membrane biosynthesis and remodeling, lipid transport and maintenance of membrane asymmetry and specific genes and protein complexes involved in OMVs, the modelSEED program was used, which integrates the biochemistry of the KEGG PATHWAY Database [110].

### 4.2. Genomic Analysis and Construction of the Pangenome Genus Klebsiella

The pangenome analysis was performed using a limited set of 11 publicly available reference genomes representing distinct species within the genus *Klebsiella*. Genomes were selected based on assembly completeness, annotation quality, and availability in public databases, rather than to capture intraspecies diversity or epidemiological variability. Consequently, clinical and environmental isolates, as well as multiple strains per species, were not included in the present analysis.

The construction of the *Klebsiella* spp. pangenome was carried out using the Compute Pangenome v0.0.7 program. The protein families present in the input genomes *Klebsiella oxytoca*, *Klebsiella African*, *Klebsiella guasivaricola*, *Klebsiella guasipneumoniae*, *Klebsiella varicola*, *Klebsiella michiganensis*, *Klebsiella pneumoiae*, *Klebsiella huaxiensis*, *Klebsiella grimontii*, *Klebsiella indica* and *Klebsiella aerogenes* were considered to form the core of the pangenome, while the non-conserved families are accessory proteins. With the KBase database, all the distinct protein families identified in a set of input genomic sequences were listed, as well as the identifiers of the proteins present in each family. This clustering allowed for a powerful set of comparisons to be made, to assess the consistency of functional assignments for highly homologous proteins, and to assess the degree to which each protein family has been conserved across the input set of genomes. The comparison of each selected genome with the pangenome was performed with the GenomeComparisonSDK v0.0.7 program [111]. This comparison method allowed us to: (I) assess the degree of gene conservation among an input set of genomes, (II) understand how genomes have evolved and adapted to their distinctive environments, and (III) identify novel genes and interesting biology in the context of related genomes.

### 4.3. Evolutionary Relationships of Proteins Associated with Horizontal Gene Transfer via Outer Membrane Vesicles in Klebsiella spp.

The taxonomic classification of the genome of *Klebsiella* species was carried out using the GTDB-Tk version 2.3.2 tool (Genome Taxonomy Database Toolkit) [112]. This software allows taxonomy assignment based on the phylogenetic analysis of whole genomes, employing a set of conserved genetic markers for accurate classification according to the assignment reliability of the GTDB database.

Similarity analysis of genes related to vesicle formation and release was performed using the Tol-cluster construct (tolABQR) and the PalA protein. For the construction of the Cluster, the selected proteins and the alignment of conserved amino acids were included, and the consensus sequence was obtained by ClustalW by using the Sequence Alignment Editor BioEdit program v 7.0.5.2, URL: https://bioedit.software.informer.com/ (accessed on 7 June 2025). Evolutionary analyzes were performed in MEGA11 [113], and the Maximum Parsimony method was used, with a boopstrap of 1000 replicates. Evolutionary distances were calculated by using the Poisson correction method based on the number of amino acid substitutions per site. The dendrogram was constructed by using the Close-Neighbor-Interchange (CNI) algorithm.

### 4.4. Genomic Analysis of Bacterial Mobilome, Resistome, and Virulome

The detection of genomic islands (GIs) and mobile genetic elements (MGEs) in *Klebsiella* genomes was performed using the IslandViewer 4 platform (http://www.pathogenomics.sfu.ca/islandviewer/) [114], which combines multiple algorithms for robust GI prediction (accessed on 23 July 2025). The following methods were used: (I) IslandPick [115], based on genomic comparison between phylogenetically related genomes, allows the identification of regions acquired through horizontal transfer, provided a sufficient number of reference genomes exist; (II) SIGI-HMM [116], which uses a hidden Markov model to detect codon usage biases and is considered one of the most accurate methods for GI prediction; and (III) IslandPath-DIMOB [115,117] which identifies candidate regions by combining dinucleotide composition analysis with the presence of mobility genes.

For the resistome analysis, the Comprehensive Antibiotic Resistance Database (CARD) [29] was used. Two complementary tools were employed: BLAST, to search for sequences homologous to known resistance genes, and the Resistance Gene Identifier (RGI), which allows for specific and accurate prediction of resistance genes. Virulence factor annotation was performed using curated databases such as VFDB [118], PATRIC [119], and Victors. For genomes without direct annotations, the Reciprocal Best BLAST Hits (RBBH) method was applied under strict criteria (e-value < 1 × 10^−10^, ≥90% identity, and ≥80% coverage). Additionally, an updated set of pathogenicity-associated genes (PAGs) was integrated [120]. Only those genes conserved in at least three pathogenic genera and absent in non-pathogenic genera were considered to ensure high functional specificity.

The results were visualized using genomic scatter plots provided by IslandViewer 4, which allowed for relative mapping of the localization of GIs, resistance genes, virulence factors, and pathogen-associated genes. Annotations were differentiated using color coding, separating direct predictions from homologous ones. Finally, the comprehensive annotation of the genome catalog included the grouping of homologous sequences to antibiotic resistance genes (RGI and CARD), pathogen-associated genes (PAGs), and virulence factors (BLAST, VFDB, PATRIC, Victors) (see Tables S4–S14.3).

## 5. Conclusions

This study provides a comprehensive comparative genomic analysis of *K. oxytoca* within the context of the *Klebsiella* genus, highlighting its remarkable genomic plasticity and its potential role as an emerging reservoir of antimicrobial resistance and virulence determinants. Through pangenome reconstruction; evolutionary analysis; and detailed characterization of the mobilome, resistome, and virulome, we propose outer membrane vesicles (OMVs) as an alternative and complementary mechanism to classical horizontal gene transfer pathways, such as conjugation and transduction.

The identification of a conserved and functionally diverse genetic repertoire associated with OMV biogenesis—including structural components, lipid and LPS remodeling pathways, and stress-responsive regulatory systems—supports the hypothesis that *K. oxytoca* possesses a robust vesiculogenic machinery capable of facilitating the dissemination of mobile genetic elements. The frequent co-localization of resistance genes, virulence factors, and mobile elements within genomic islands further reinforces the idea that this species represents a genomic hotspot for adaptive evolution under strong selective pressures, particularly in hospital environments.

However, this work is based primarily on in silico comparative genomic analyses using publicly available reference genomes, which constitutes an important limitation. The absence of direct clinical or environmental metagenomic samples, as well as the lack of experimental validation, means that the proposed OMV-mediated transfer mechanisms should be interpreted as theoretical and predictive models, rather than direct functional demonstrations. In addition, the study does not address microbial community dynamics or ecological interactions that may influence OMV production and gene transfer in real-world microbiomes.

Future research should therefore focus on integrating functional and experimental approaches, including OMV isolation, molecular cargo characterization, and transfer assays, as well as metagenomic and metatranscriptomic analyses of clinical and environmental samples. Such studies will be essential to validate the biological relevance of OMV-mediated gene transfer and to assess its impact on the dissemination of antimicrobial resistance at the community level. Additionally, longitudinal studies evaluating OMV dynamics under antibiotic pressure could help translate these genomic insights into practical strategies for infection control, surveillance, and antimicrobial stewardship.

In conclusion, although the present study is exploratory and hypothesis-driven, it establishes a solid genomic framework that positions *K. oxytoca* as a species of high evolutionary and clinical relevance. Understanding OMV-mediated genetic exchange may open new avenues for monitoring bacterial adaptation and for developing innovative strategies to limit the spread of resistance and virulence in clinical microbiomes.

## Figures and Tables

**Table 1:** *Klebsiella* genomes included in the pangenome analysis.

Species	Accession ^a^	Genome Size (Mb)	Total Genes	Protein-Coding Genes	Genes in Homologous Clusters	Singleton Genes ^b^	Homologous Gene Families ^c^
*K. oxytoca*	GCF_001022115.1	6.7	6286	5276	5679	607	5204
*K. africana*	GCA_016804125.1	5.4	5203	4982	4812	269	4585
*K. quasivariicola*	GCF_002269255.1	5.9	6064	5703	5366	382	4900
*K. quasipneumoniae*	GCF_001278905.1	5.1	4852	4614	4602	250	4402
*K. variicola*	GCF_000828055.2	5.5	5258	4992	4912	346	4673
*K. michiganensis*	GCF_000783895.2	6.5	6457	6015	5523	713	5099
*K. pneumoniae*	GCA_000764615.1	6.0	5812	5594	5135	677	4790
*K. huaxiensis*	GCF_003261575.2	6.3	6048	5716	5073	753	4709
*K. grimontii*	GCF_013590775.1	6.5	6345	6010	5569	644	5071
*K. indica*	GCF_005860775.1	5.2	4920	4632	3966	694	3708
*K. aerogenes*	GCF_000755545.1	5.1	4795	4692	4110	671	3906

^a^—Accession numbers correspond to NCBI entries. ^b^—Singleton genes correspond to strain-specific. ^c^—Homologous gene families include core, soft-core, and accessory clusters defined by the pangenome analysis. Mb, megabases.

**Table 2:** Comparative analysis of homologous gene families among *Klebsiella* species.

Genome *	G1	G2	G3	G4	G5	G6	G7	G8	G9	G10	G11
G1	–	3483	3428	3391	3027	3495	3509	3489	3498	3477	3531
G2	3483	–	3780	3650	3210	3819	3804	4234	4008	4257	4177
G3	3428	3780	–	4083	3406	4437	4460	3830	3663	3850	3790
G4	3391	3650	4083	–	3457	4151	4214	3680	3594	3722	3674
G5	3027	3210	3406	3457	–	3493	3408	3212	3208	3210	3267
G6	3495	3819	4437	4151	3493	–	4406	3886	3741	3874	3854
G7	3509	3804	4460	4214	3408	4406	–	3878	3709	3851	3764
G8	3489	4234	3830	3680	3212	3886	3878	–	4090	4234	4184
G9	3498	4008	3663	3594	3208	3741	3709	4090	–	3979	4235
G10	3477	4257	3850	3722	3210	3874	3851	4234	3979	–	4225
G11	3531	4177	3790	3674	3267	3854	3764	4184	4235	4225	–

* G1: K. aerogenes, G2: K. africana, G3: K. grimontii, G4: K. huaxiensis, G5: K. indica, G6: K. michiganensis, G7: K. oxytoca, G8: K. pneumoniae, G9: K. quasipneumoniae, G10: K. quasivariicola, G11: K. variicola. Values indicate the number of shared homologous gene families identified between genome pairs based on orthology clustering. Diagonal cells (–) indicate self-comparisons. Genome abbreviations correspond to the species listed in the header. This comparative analysis provides a quantitative measure of genomic relatedness across the Klebsiella genus.

**Table 3:** Genes and protein complexes associated with outer membrane structure and stress response potentially involved in outer membrane vesicle-mediated extracellular genetic material transfer in *Klebsiella oxytoca*.

Gene	Protein Name	Accession Number	Gene/Protein Length	Associated Complex or System	Annotated Biological Function ^a^	Phenotype Reported in Gram-Negative Bacteria	Interpretation Related to OMV/EGM ^b^
ompA	Outer membrane protein A	WP_014226437.1	483 bp/160 aa	OM–PG linker	Outer membrane porin involved in maintaining outer membrane–peptidoglycan interactions	Reduced envelope stability and increased membrane permeability upon loss	Envelope destabilization may favor OMV release and extracellular DNA packaging
bamA	BAM complex subunit A	WP_004107929.1	2439 bp/812 aa	BAM complex	Insertion and assembly of β-barrel outer membrane proteins	Accumulation of misfolded OM proteins	Envelope stress is correlated with increased OMV production
bamB	BAM accessory protein	WP_004104478.1	1179 bp/392 aa	BAM complex	Facilitates β-barrel protein assembly	Reduced efficiency of OM protein insertion	Defective assembly may indirectly enhance vesiculation
tolA	Tol-Pal system protein TolA	WP_032748630.1	936 bp/311 aa	Tol–Pal complex	Maintains physical connectivity of the cell envelope	OM detachment	Loss of envelope cohesion may enhance OMV biogenesis
tolB	Tol-Pal system protein TolB	WP_004130584.1	1293 bp/430 aa	Tol–Pal complex	Periplasmic scaffold protein of the Tol–Pal system	Increased vesiculation	Enhanced OMV formation may facilitate EGM release
pal	Peptidoglycan-associated lipoprotein	WP_014228456.1	693 bp/230 aa	Tol–Pal complex	Anchors outer membrane to peptidoglycan	Weakened OM attachment	Reduced anchoring may promote OMV release
nlpI	Outer membrane lipoprotein NlpI	WP_004854535.1	885 bp/294 aa	Envelope regulatory	Regulates proteolysis and envelope homeostasis	Envelope stress response activation	Stress-associated conditions correlate with increased OMV production
surA	Periplasmic chaperone SurA	WP_004098530.1	1287 bp/428 aa	Chaperone network	Assists folding of outer membrane proteins	Accumulation of misfolded proteins	Protein misfolding stress may induce OMV release
lpp	Murein lipoprotein	WP_003021624.1	624 bp/207 aa	OM–PG tether	Structural outer membrane–peptidoglycan linkage	Reduced envelope rigidity	Envelope weakening may increase vesiculation
degP	Serine protease/chaperone DegP	WP_004098740.1	1440 bp/479 aa	Periplasmic QC	Degradation of misfolded periplasmic proteins	Activation of envelope stress response	Stress response is associated with elevated OMV levels
mlaA	Mla pathway protein A	WP_014230289.1	762 bp/253 aa	Mla system	Maintains outer membrane lipid asymmetry	Phospholipid accumulation in outer leaflet	Altered lipid asymmetry may promote membrane curvature
msbA	ABC transporter MsbA	WP_032748909.1	1749 bp/582 aa	LPS transport	Translocation of lipopolysaccharide	LPS accumulation in inner membrane	Lipid imbalance may enhance OMV formation
pmrA/B	Two-component system PmrA/B	WP_004849045.1	672 bp/223 aa	PmrAB system	Regulation of envelope modification genes	Altered lipid A composition	Envelope remodeling may indirectly modulate OMV biogenesis
phoP/Q	Two-component system PhoP/Q	WP_004101181.1	675 bp/224 aa	PhoPQ system	Response to Mg^2+^ limitation and antimicrobial peptides	Membrane remodeling and stress adaptation	Stress-induced remodeling may favor OMV production

OM, outer membrane; PG, peptidoglycan; OMV, outer membrane vesicle; EGM, extracellular genetic material. ^a^—Annotated biological functions are based on curated descriptions and conserved mechanisms in Gram-negative bacteria. ^b^—Interpretations represent evidence-based associations rather than direct causal relationships.

## Data Availability

The original contributions presented in this study are included in the article/Supplementary Materials. Further inquiries can be directed to the corresponding author(s).

## Supplementary Materials

The following supporting information can be downloaded from https://www.mdpi.com/article/10.3390/ijms27020988/s1.

## Abbreviations

The following abbreviations are used in this manuscript:

AMR	Antimicrobial resistance
ABC	ATP-binding cassette
AraC	Arabinose-responsive transcriptional regulator
CRP	cAMP receptor protein
DMT	Drug/metabolite transporter
DNA	Deoxyribonucleic acid
EV	Extracellular vesicle
GI	Genomic island
HGT	Horizontal gene transfer
ICE	Integrative and conjugative element
IS	Insertion sequence
LPS	Lipopolysaccharide
MFS	Major facilitator superfamily
MGE	Mobile genetic element
OMV	Outer membrane vesicle
PBP	Penicillin-binding protein
R-M	Restriction–modification
RND	Resistance–nodulation–cell division
SOS	DNA damage–induced stress response system
TA	Toxin–antitoxin
T4SS	Type IV secretion system
T6SS	Type VI secretion system
