# Supplementary material for: A Possible Recently Identified Evolutionary Strategy Using Membrane-Bound Vesicle Transfer of Genetic Material to Induce Bacterial Resistance, Virulence and Pathogenicity in Klebsiella oxytoca
Source: Int J Mol Sci. 2026 Jan 19;27(2):988. doi: 10.3390/ijms27020988 (PMC12842022; doi:10.3390/ijms27020988)
Supplement: Supplementary file 1 [file ijms-27-00988-s001.zip › Figures supplementary S1-S5.pdf]

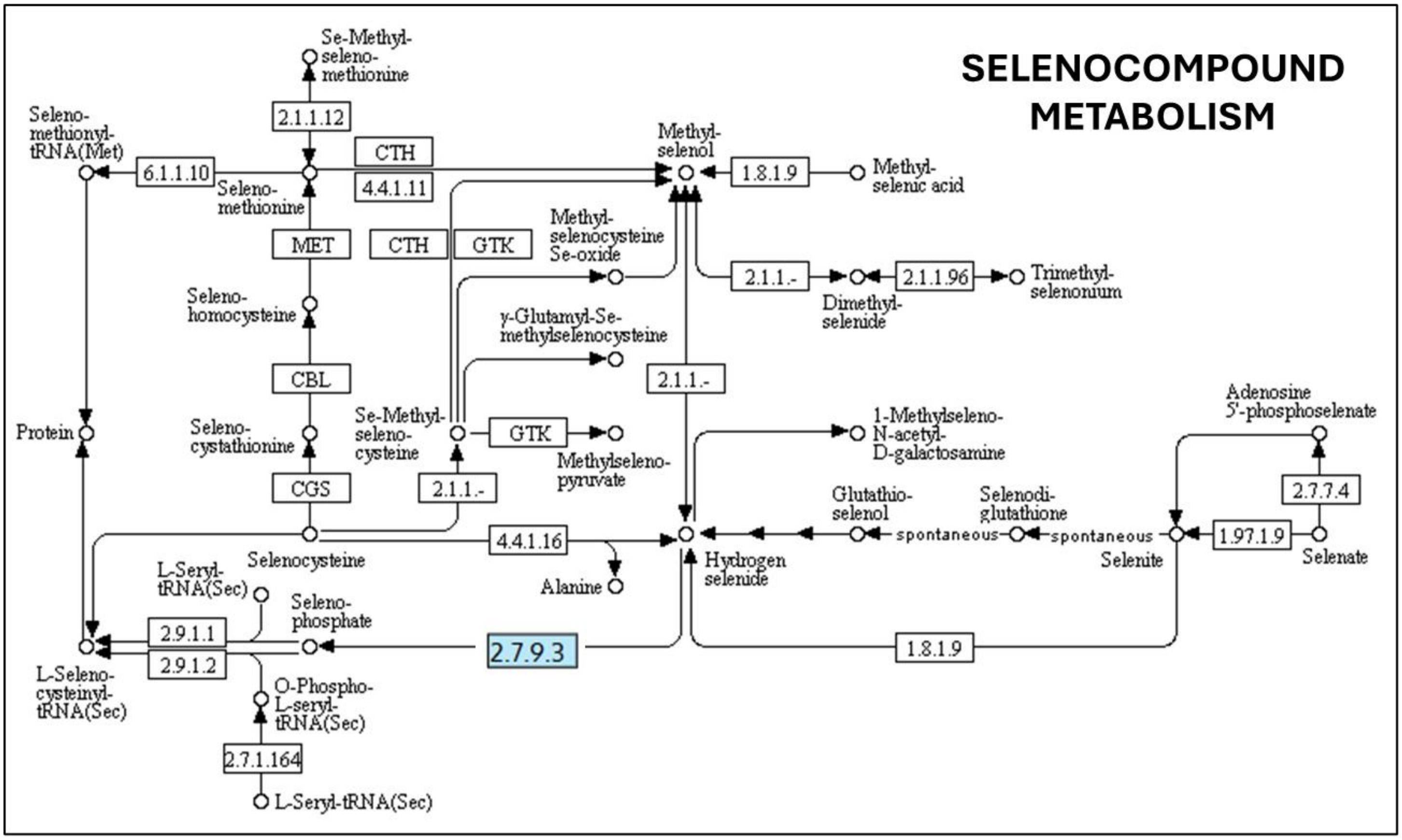

Figure S1. Selenocompound metabolic pathway in *Klebsiella oxytoca*. The diagram shows the enzymatic steps involved in selenocompound metabolism according to the KEGG database. Of all the genes comprising this pathway, only the one corresponding to the enzyme EC 2.7.9.3 (selenophosphate synthase), indicated in color, was identified in the analyzed genome. This enzyme catalyzes the conversion of ATP and selenite into selenophosphate, an essential intermediate for selenoprotein biosynthesis. The absence of other genes in the pathway suggests a limited metabolic capacity for the complete processing of selenocompounds in this bacterial strain.

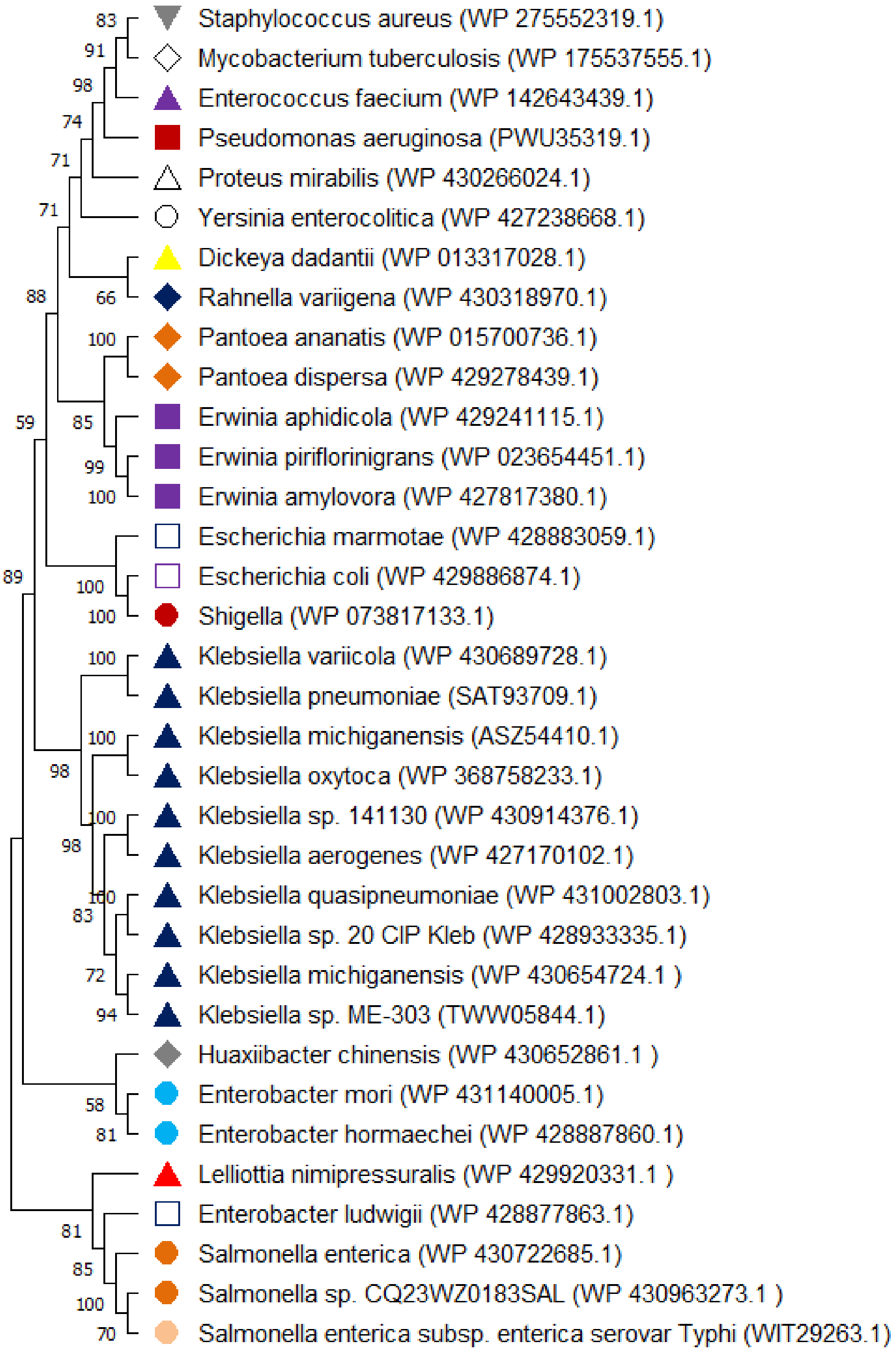

Figure S2. Phylogenetic trees based on aminoacid sequences of TolA in bacteria. Phylogeny was reconstructed by Maximum Parsimony and cluster confidence was tested by 1000 bootstrap iterations. The amino acid sequences were aligned with Aligment Explorer/CLUSTALW program and the software Genetic and Molecular Evolution Analyses (MEGA 11).

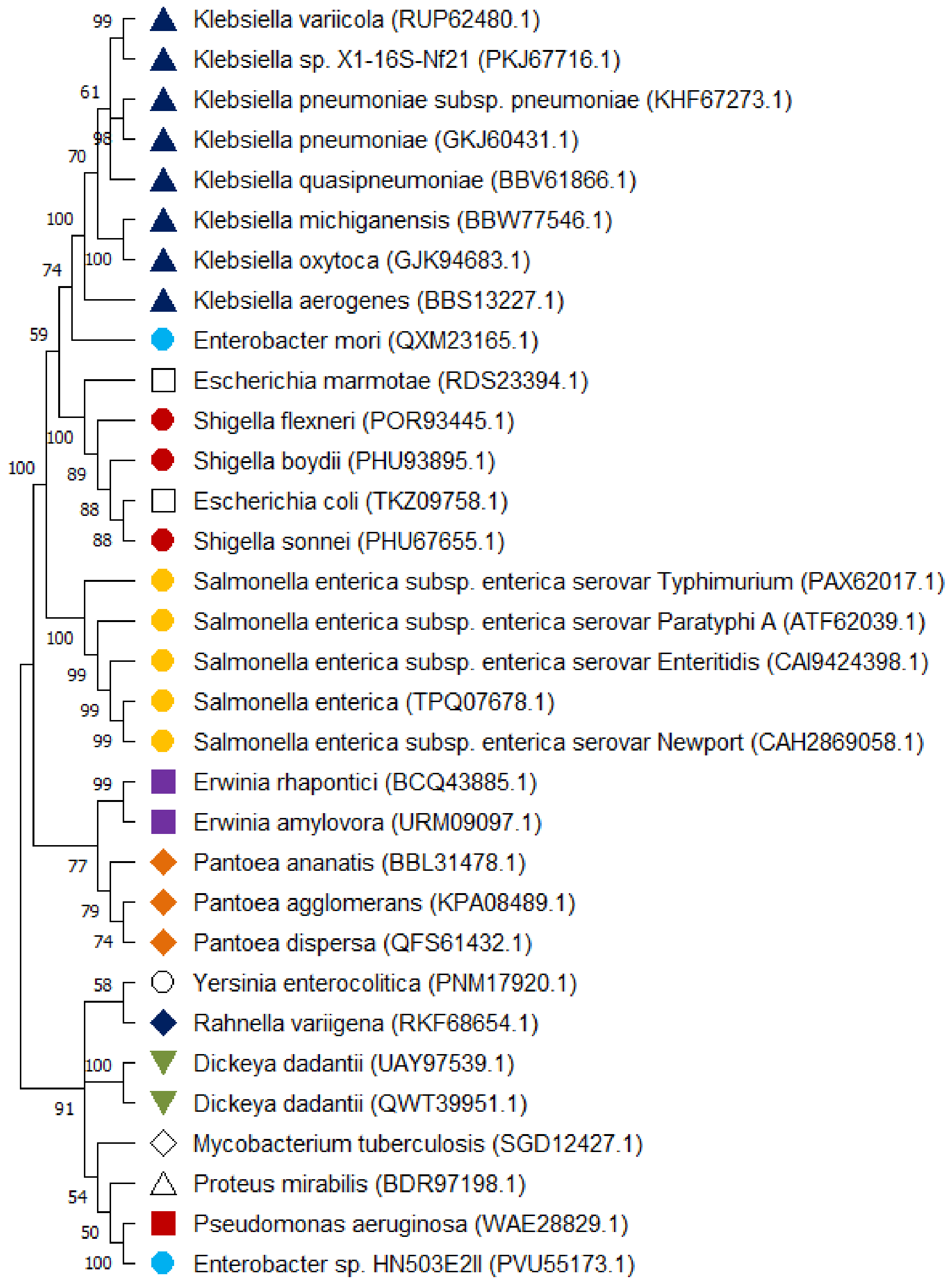

Figure S3. Phylogenetic trees based on aminoacid sequences of TolB in bacteria. Phylogeny was reconstructed by Maximum Parsimony and cluster confidence was tested by 1000 bootstrap iterations. The amino acid sequences were aligned with Aligment Explorer/CLUSTALW program and the software Genetic and Molecular Evolution Analyses (MEGA 11).

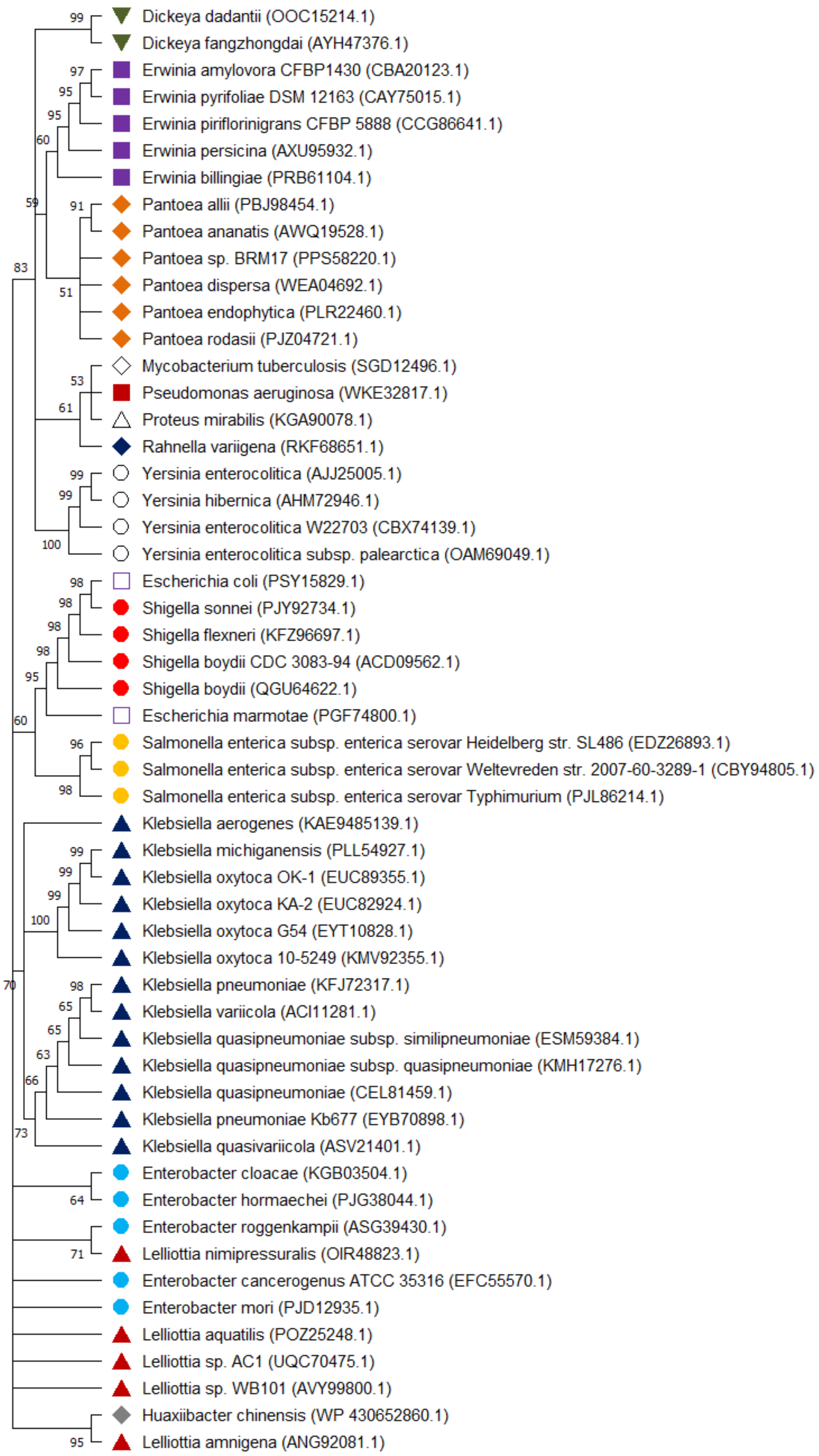
Dickeya dadantii (OOC15214.1)
Dickeya fangzhongdai (AYH47376.1)
Erwinia amylovora CFBP1430 (CBA20123.1)
Erwinia pyrifoliae DSM 12163 (CAY75015.1)
Erwinia piriflorinigrans CFBP 5888 (CCG86641.1)
Erwinia persicina (AXU95932.1)
Erwinia billingiae (PRB61104.1)
Pantoea allii (PBJ98454.1)
Pantoea ananatis (AWQ19528.1)
Pantoea sp. BRM17 (PPS58220.1)
Pantoea dispersa (WEA04692.1)
Pantoea endophytica (PLR22460.1)
Pantoea rodasii (PJZ04721.1)
Mycobacterium tuberculosis (SGD12496.1)
Pseudomonas aeruginosa (WKE32817.1)
Proteus mirabilis (KGA90078.1)
Rahnella variigena (RKF68651.1)
Yersinia enterocolitica (AJJ25005.1)
Yersinia hibernica (AHM72946.1)
Yersinia enterocolitica W22703 (CBX74139.1)
Yersinia enterocolitica subsp. palearctica (OAM69049.1)
Escherichia coli (PSY15829.1)
Shigella sonnei (PJY92734.1)
Shigella flexneri (KFZ96697.1)
Shigella boydii CDC 3083-94 (ACD09562.1)
Shigella boydii (QGU64622.1)
Escherichia marmotae (PGF74800.1)
Salmonella enterica subsp. enterica serovar Heidelberg str. SL486 (EDZ26893.1)
Salmonella enterica subsp. enterica serovar Weltevreden str. 2007-60-3289-1 (CBY94805.1)
Salmonella enterica subsp. enterica serovar Typhimurium (PJL86214.1)
Klebsiella aerogenes (KAE9485139.1)
Klebsiella michiganensis (PLL54927.1)
Klebsiella oxytoca OK-1 (EUC89355.1)
Klebsiella oxytoca KA-2 (EUC82924.1)
Klebsiella oxytoca G54 (EYT10828.1)
Klebsiella oxytoca 10-5249 (KMV92355.1)
Klebsiella pneumoniae (KFJ72317.1)
Klebsiella variicola (ACI11281.1)
Klebsiella quasipneumoniae subsp. similipneumoniae (ESM59384.1)
Klebsiella quasipneumoniae subsp. quasipneumoniae (KMH17276.1)
Klebsiella quasipneumoniae (CEL81459.1)
Klebsiella pneumoniae Kb677 (EYB70898.1)
Klebsiella quasivariicola (ASV21401.1)
Enterobacter cloacae (KGB03504.1)
Enterobacter hormaechei (PJG38044.1)
Enterobacter roggenkampii (ASG39430.1)
Lelliottia nimipressuralis (OIR48823.1)
Enterobacter cancerogenus ATCC 35316 (EFC55570.1)
Enterobacter mori (PJD12935.1)
Lelliottia aquatilis (POZ25248.1)
Lelliottia sp. AC1 (UQC70475.1)
Lelliottia sp. WB101 (AVY99800.1)
Huaxiibacter chinensis (WP 430652860.1)
Lelliottia amnigena (ANG92081.1)

Figure S4. Phylogenetic trees based on aminoacid sequences of TolQ in bacteria. Phylogeny was reconstructed by Maximum Parsimony and cluster confidence was tested by 1000 bootstrap iterations. The amino acid sequences were aligned with Aligment Explorer/CLUSTALW program and the software Genetic and Molecular Evolution Analyses (MEGA 11).

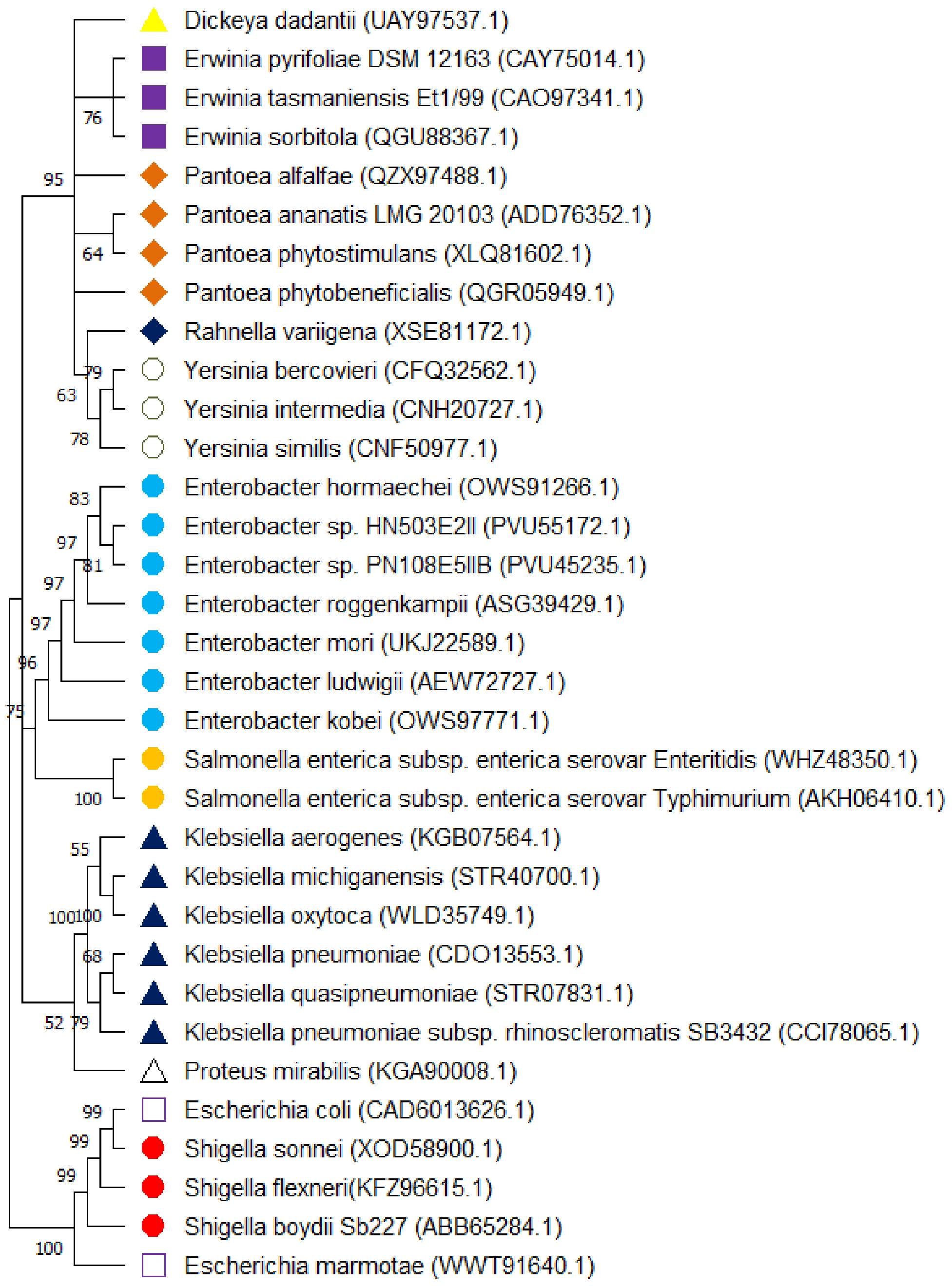

Figure S5. Phylogenetic trees based on aminoacid sequences of TolR in bacteria. Phylogeny was reconstructed by Maximum Parsimony and cluster confidence was tested by 1000 bootstrap iterations. The amino acid sequences were aligned with Aligment Explorer/CLUSTALW program and the software Genetic and Molecular Evolution Analyses (MEGA 11).
