# Supplementary material for: A Possible Recently Identified Evolutionary Strategy Using Membrane-Bound Vesicle Transfer of Genetic Material to Induce Bacterial Resistance, Virulence and Pathogenicity in Klebsiella oxytoca
Source: Int J Mol Sci. 2026 Jan 19;27(2):988. doi: 10.3390/ijms27020988 (PMC12842022; doi:10.3390/ijms27020988)
Supplement: Supplementary file 1 [file ijms-27-00988-s001.zip › Tables supplementary 1., 2..pdf]

Table S1. Functional Genes in *K. oxytoca* Related to OMV Formation and Release

Functional Association	Gene	Enzyme Name	EC Number	KO (KEGG Orthology)	Substrate	Main Function	Implication in OMVs Formation
			Biosynthesis and remodeling of the outer membrane				
Lipopolysaccharide biosynthesis	LpXA	UDP-N-acetylglucosamine acyltransferase	2.3.1.129	K02535	UDP-GlcNAc, Acyl-ACP	Initiates lipid A biosynthesis of LPS	Structural changes in lipid A affect curvature and OMV release
	LpXB	Lipid-A-disaccharide synthase	2.4.1.182	K02539	GlcN(ACYL)-P + UDP-GlcN(ACYL)	Assembles lipid A disaccharide backbone	Affects outer membrane bilayer integrity
	LpXC	UDP-3-O-acyl N-acetylglucosamine deacetylase	3.5.1.108	K02536	UDP-3-O-acyl-GlcNAc	Rate-limiting step in lipid A biosynthesis	Regulates LPS accumulation; impacts OMV formation
	LpXD	UDP-3-O-(acyl)-GlcN N-acyltransferase	2.3.1.191	K02537	UDP-3-O-acyl-GlcN	Adds second acyl group	Determines lipid hydrophobicity; relates to OMV release
	LpXH	UDP-2,3-diacylglucosamine hydrolase	3.6.1.54	K02538	UDP-2,3-diacyl-GlcN	Hydrolyzes UDP to form lipid A precursor	Fundamental precursor for structural LPS
	LpXL	Lauryl acyltransferase	2.3.1.241	K04744	Lipid A	Adds laurate to lipid A	Alters membrane fluidity and vesicle release
	LpXK	Tetraacyldisaccharide 4'-kinase	2.7.1.130	K02540	Lipid A disaccharide	Phosphorylates to stabilize diphosphate lipid A	Critical for LPS anchoring and membrane stability

	LpXM	Myristoyl transferase	2.3.1.243	K04745	Lipid A	Adds myristate to lipid A	Increases LPS hydrophobicity, associated with vesiculation
	KdtA (WaaA)	KDO transferase	2.4.99.12	K00920	Lipid A + KDO	Adds KDO to lipid A, linking to LPS core	KDO modifications impact immunogenicity and OMV formation
	GmhA	Sedoheptulose-7-phosphate isomerase	5.3.1.28	K03273	Sedoheptulose-7P	Initiates heptose biosynthesis for LPS core	Heptose affects LPS core stability and OMV formation
	GmhC (H1dE)	Bifunctional heptose kinase and ADP-transferase	2.7.1.167 / 2.7.7.70	K03274	D-glycero-D-manno-heptose-7P	Activates heptose for LPS core incorporation	Modulates LPS structure, impacting OMV packaging
	GmhB (YaeD)	D-glycero-D-manno-heptose 1,7-bisphosphate phosphatase	3.1.3.82	K03272	D-glycero-D-manno-heptose 1,7-P2	Dephosphorylates heptose intermediate in LPS biosynthesis	Essential for heptose flow to LPS core; defects may enhance OMV formation
	GmhD (RfaD)	ADP-L-glycero-D-manno-heptose 6-epimerase	5.1.3.20	K03275	ADP-heptose	Produces active heptose form for LPS core	Contributes to LPS structural integrity and vesicle biogenesis
Peptidoglycan biosynthesis	murA	UDP-N-acetylglucosamine 1-carboxyvinyltransferase	2.5.1.7	K00790	UDP-GlcNAc, phosphoenolpyruvate	First step in peptidoglycan biosynthesis	Disruption affects envelope integrity; promotes OMVs

murB	UDP-N-acetylenolpyruvoylglucosamine reductase	1.3.1.98	K00075	UDP-N-acetylglucosamine-enolpyruvate	Reduction to lactyl group precursor	Alters precursor pool; may affect OMV release
murC	UDP-N-acetylmuramoylalanine synthetase	6.3.2.8	K01923	UDP-MurNAc, L-alanine	Adds L-Ala to peptidoglycan precursor	Involved in cell wall assembly; wall defects linked to OMVs
murD	UDP-N-acetylmuramoyl-L-alanine-D-glutamate ligase	6.3.2.9	K01924	UDP-MurNAc-L-Ala, D-Glu	Adds D-Glu to peptide chain	Modifies rigidity; imbalance may induce vesiculation
ddl	D-alanine--D-alanine ligase	6.3.2.4	K01921	2 D-alanine	Forms D-Ala-D-Ala dipeptide for cross-linking	Impacts crosslinking and wall tension
murE	UDP-MurNAc-tripeptide synthetase	6.3.2.13	K01925	UDP-MurNAc-L-Ala-D-Glu, meso-DAP or L-Lys	Adds third amino acid to stem peptide	Disruption changes peptide bridge formation and wall structure
murF	UDP-N-acetylmuramoyl-pentapeptide synthetase	6.3.2.10	K01926	UDP-MurNAc-tripeptide, D-Ala-D-Ala	Completes pentapeptide precursor	Defects affect peptidoglycan structure and envelope integrity
mraY	UDP-MurNAc-pentapeptide phospho-N-acetylmuramoyltransferase (MraY)	2.7.8.13	K00791	UDP-MurNAc-pentapeptide,	Initiates lipid-linked peptidoglycan synthesis	Links cytosolic steps to membrane, key in OMVs regulation

					undecaprenyl phosphate		
	murG	UDP-N-acetylglucosamine transferase	2.4.1.227	K00793	Lipid I, UDP-GlcNAc	Forms Lipid II (final PG precursor)	Lipid II imbalance affects membrane tension and OMV production
	uppP	Undecaprenyl-diphosphatase (BacA)	3.6.1.27	K06147	Undecaprenyl diphosphate	Recycles lipid carrier for peptidoglycan biosynthesis	Carrier limitation impacts cell wall homeostasis and OMV release
Glycerophospholipid metabolism	gpsA	Glycerol-3-phosphate dehydrogenase (NAD^+)	1.1.1.8	K00057	Dihydroxyacetone phosphate (DHAP), NADH	Converts DHAP to glycerol-3-phosphate (G3P), key for phospholipid biosynthesis	Essential precursor for membrane glycerophospholipids; affects OMV formation
	glpD	Glycerol-3-phosphate dehydrogenase (quinone)	1.1.5.3	K00111	Glycerol-3-phosphate, quinone	Catalyzes oxidation of G3P to DHAP in respiration	Alters G3P levels, impacts membrane fluidity and OMV release
	plsB	Glycerol-3-phosphate acyltransferase	1.1.1.94	K00691	G3P, acyl-ACP	First step in phosphatidic acid synthesis	Initiates phospholipid synthesis; imbalances influence OMV composition

	plsC	1-acyl-sn-glycerol-3-phosphate acyltransferase	1.1.1.261	K00692	1-acyl-G3P, acyl-ACP	Forms phosphatidic acid from 1-acyl-G3P	Critical for membrane phospholipids; may modulate OMVs lipid profile
Selenocompund metabolism	selD	Selenide, water dikinase	2.7.9.3	K00860	Selenide, ATP	Converts selenide into selenophosphate, for selenoprotein biosynthesis	Potential oxidative stress regulator; stress promotes OMVs as detox mechanism

Table S2. Antibiotic Resistance Genes (AMR) Associated with Outer Membrane Vesicles (OMVs)

Gene ARO	AMR Gene family	Drug Class	Resistance Mechanism	Role in Resistance	Evidence of Expression in OMVs	HGT Mechanism
KpnE	SMR efflux pump	Macrolide antibiotic, aminoglycoside antibiotic, cephalosporin, tetracycline antibiotic, peptide antibiotic, rifamycin antibiotic, disinfecting agents and antiseptics	Antibiotic efflux	Pumps antibiotics out of the cell	Detected in OMV proteomics of K. pneumoniae [119,33]	Gene transfer via OMVs protecting recipient cells
KpnF	SMR efflux pump	Macrolide antibiotic, aminoglycoside antibiotic, cephalosporin, tetracycline antibiotic, peptide antibiotic, rifamycin antibiotic, disinfecting agents and antiseptics	Antibiotic efflux	Similar efflux pump function	OMV proteomics [6,135*]	OMVs as genetic transfer vehicles
qacJ	SMR efflux pump	Disinfecting agents and antiseptics	Antibiotic efflux	Expels toxic compounds	OMVs of Klebsiella and other Enterobacteriaceae [6,120]	Transfer via OMVs facilitates collective resistance
marA	RND efflux + Porin with reduced permeability to beta-lactams	Fluoroquinolone antibiotic, monobactam, carbapenem,	Antibiotic efflux, reduced	Regulates efflux pumps and porins	Expression detected in OMVs [121,26]	Indirect resistance

		cephalosporin, glycylcycline, penicillin beta-lactam, tetracycline antibiotic, rifamycin antibiotic, phenicol antibiotic, disinfecting agents and antiseptics	permeability to antibiotic			transfer via OMVs
adeF	RND efflux pump	Fluoroquinolone antibiotic, tetracycline antibiotic	Antibiotic efflux	Active efflux pump	Indirect evidence by homology with OMV-expressed systems [120]	OMVs facilitate transfer of RND genes
baeR	RND efflux pump	Aminoglycoside antibiotic, aminocoumarin antibiotic	Antibiotic efflux	Efflux regulator	Partial proteomic detection in OMVs [135*]	OMVs in transfer of resistance regulators
CRP	RND efflux pump	macrolide antibiotic, fluoroquinolone antibiotic, penicillin beta-lactam	Antibiotic efflux	Controls resistance genes	Expression observed in OMVs [119]	Gene transfer facilitated by OMVs
AcrAB-TolC	RND efflux pump	Fluoroquinolone antibiotic, cephalosporin, glycylcycline, penicillin beta-lactam, tetracycline antibiotic, rifamycin antibiotic, phenicol antibiotic, disinfecting agents and antiseptics	Antibiotic target alteration, antibiotic efflux	Main efflux system	Abundantly identified in Klebsiella OMVs [120,6]	OMVs carry AcrAB-TolC genes and proteins

oqxA	RND efflux pump	Fluoroquinolone antibiotic, glycylcycline, tetracycline antibiotic, diaminopyrimidine antibiotic, nitrofuran antibiotic	Antibiotic efflux	Active antibiotic extrusion	Detected in bacterial OMVs [120]	OMVs contribute to oqxA dissemination
rsmA	RND efflux pump	fluoroquinolone antibiotic, diaminopyrimidine antibiotic, phenicol antibiotic	Antibiotic efflux	Regulator and efflux pump	Detected in membrane vesicles [121*]	Likely transfer by OMVs
acrA	RND efflux pump	Fluoroquinolone antibiotic, cephalosporin, glycylcycline, penicillin beta-lactam, tetracycline antibiotic, rifamycin antibiotic, phenicol antibiotic, disinfecting agents and antiseptics	Antibiotic efflux	Part of AcrAB-TolC system	Proteomics confirms presence in OMVs [6]	Transfer via membrane vesicles
H-NS	MFS and RND efflux	Macrolide antibiotic, fluoroquinolone antibiotic, cephalosporin, penicillin beta-lactam, tetracycline antibiotic	Antibiotic efflux	Global regulator modulating pumps	Reported in OMVs [121]	OMVs can transfer regulatory factors
emrR	major facilitator superfamily (MFS) antibiotic efflux pump	Fluoroquinolone antibiotic	Antibiotic efflux	Efflux regulator	Limited evidence in OMVs, based on homology [120]	OMVs possible genetic vehicles
KpnG	MFS efflux pump	Macrolide antibiotic, fluoroquinolone	Antibiotic efflux	Efflux pump	OMV proteomics in Klebsiella [119]	Transfer via vesicles

		antibiotic, aminoglycoside antibiotic, carbapenem, cephalosporin, penicillin beta-lactam, peptide antibiotic				
KpnH	MFS efflux pump	Macrolide antibiotic, fluoroquinolone antibiotic, aminoglycoside antibiotic, carbapenem, cephalosporin, penicillin beta-lactam, peptide antibiotic	Antibiotic efflux	Similar to KpnG	Expression detected in OMVs [6]	Genetic transfer by OMVs
leuO	MFS efflux pump	Nucleoside antibiotic, disinfecting agents and antiseptics	Antibiotic efflux	Regulator and efflux pump	Reported in OMVs [120]	OMVs facilitate transfer
LptD	ABC efflux pump	carbapenem, peptide antibiotic, aminocoumarin antibiotic, rifamycin antibiotic	Antibiotic efflux	Active transporter	Proteomic evidence in OMVs [135*]	OMVs carry transporters
msbA	ABC efflux pump	Nitroimidazole antibiotic	Antibiotic efflux	Active transporter	Detected in OMVs [121]	Transfer by vesicles
ArnT	pmr phosphoethanolamine transferase	Peptide antibiotic	Antibiotic target alteration	LPS modification for resistance	Detected in membranes and OMVs [119]	OMVs can transfer LPS modifications
eptB	pmr phosphoethanolamine transferase	Peptide antibiotic	Antibiotic target alteration	Similar to ArnT	Indirect evidence in OMVs [120]	Transfer via vesicles possible

PBP3	Penicillin-binding protein mutations	Cephalosporin, penicillin beta-lactam	Antibiotic target alteration	Altered antibiotic target site	Not common in OMVs; gene transfer mainly via plasmids [122]	More frequent plasmid-mediated transfer
vanG	Van ligase (glycopeptide resistance)	Glycopeptide antibiotic	Antibiotic target alteration	Modifies D-Ala-D-Lac	Not reported in OMVs in Klebsiella [122]	Transfer by mobile elements
gyrB	Fluoroquinolone resistant gyrB	Fluoroquinolone antibiotic	Antibiotic target alteration	DNA gyrase mutation	Not reported in OMVs [122]	Genomic transfer
OXY-2-2	OXY beta-lactamase	Monobactam, cephalosporin, penicillin beta-lactam	antibiotic inactivation	Hydrolyzes beta-lactams	Beta-lactamases detected in OMVs [120]	OMVs release enzymes for community protection
fosA5	Fosfomycin thiol transferase	fluoroquinolone antibiotic, aminoglycoside antibiotic, phosphonic acid antibiotic	antibiotic inactivation	Enzymatic inactivation	OMV proteomics [119]	OMVs carry enzymes
OmpA	Porin with reduced permeability	Peptide antibiotic	Reduced permeability to antibiotic	Physical barrier and regulation	Highly present in OMVs [120]	OMVs carry porins to modulate resistance

**[135] Brown, L., Wolf, J. M., Prados-Rosales, R. & Casadevall, A. Through the wall: Extracellular vesicles in Gram-positive bacteria, mycobacteria and fungi. Nat. Rev. Microbiol. 13, 620–630. https://doi.org/10.1038/nrmicro3480 (2015).*
